# Engineering Metal Halide Perovskite Nanocrystals with BODIPY Dyes for Photosensitization and Photocatalytic Applications

**DOI:** 10.1021/jacs.3c14335

**Published:** 2024-04-04

**Authors:** Alejandro Cortés-Villena, Delia Bellezza, Carla Cunha, Ignacio Rosa-Pardo, Álvaro Seijas-Da Silva, João Pina, Gonzalo Abellán, J. Sérgio Seixas de Melo, Raquel E. Galian, Julia Pérez-Prieto

**Affiliations:** † Institute of Molecular Science, University of Valencia, c/Catedrático José Beltrán Martínez 2, 46980 Paterna, Valencia, Spain; ‡ CQC-IMS, Department of Chemistry, University of Coimbra, Coimbra P-3004-535, Portugal

## Abstract

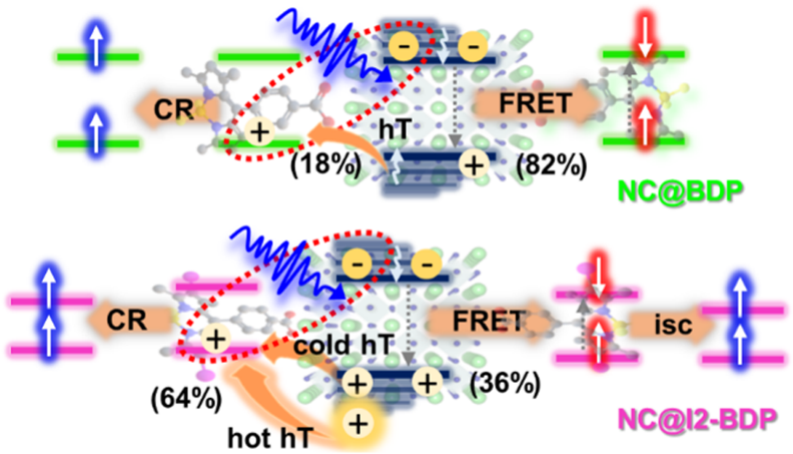

The sensitization of surface-anchored organic dyes on semiconductor nanocrystals through energy transfer mechanisms has received increasing attention owing to their potential applications in photodynamic therapy, photocatalysis, and photon upconversion. Here, we investigate the sensitization mechanisms through visible-light excitation of two nanohybrids based on CsPbBr_3_ perovskite nanocrystals (NC) functionalized with borondipyrromethene (BODIPY) dyes, specifically 8-(4-carboxyphenyl)-1,3,5,7-tetramethyl-4,4-difluoro-4-bora-3a,4a-diaza-s-indacene (BDP) and 8-(4-carboxyphenyl)-2,6-diiodo-1,3,5,7-tetramethyl-4,4-difluoro-4-bora-3a,4a-diaza-s-indacene (I2-BDP), named as NC@BDP and NC@I2-BDP, respectively. The ability of I2-BDP dyes to extract hot hole carriers from the perovskite nanocrystals is comprehensively investigated by combining steady-state and time-resolved fluorescence as well as femtosecond transient absorption spectroscopy with spectroelectrochemistry and quantum chemical theoretical calculations, which together provide a complete overview of the phenomena that take place in the nanohybrid. Förster resonance energy transfer (FRET) dominates (82%) the photosensitization of the singlet excited state of BDP in the NC@BDP nanohybrid with a rate constant of 3.8 ± 0.2 × 10^10^ s^–1^, while charge transfer (64%) mediated by an ultrafast charge transfer rate constant of 1.00 ± 0.08 × 10^12^ s^–1^ from hot states and hole transfer from the band edge is found to be mainly responsible for the photosensitization of the triplet excited state of I2-BDP in the NC@I2-BDP nanohybrid. These findings suggest that the NC@I2-BDP nanohybrid is a unique energy transfer photocatalyst for oxidizing α-terpinene to ascaridole through singlet oxygen formation.

## Introduction

The new era of advanced materials envisions perovskites as emerging semiconductor materials for optoelectronics technology and energy conversion applications.^1,2^ Merging semiconductor nanocrystals or quantum dots (QDs) with functional organic molecules has been used as a strategy to produce novel hybrid nanomaterials, which not only leverage the advantages of each component but also unlock new capabilities, such as light harvesting.^3^ Proper photon harvesting is one of the key issues in designing an efficient hybrid material.^4^ In particular, energy transfer (ET) processes are of special interest precisely because they mimic the processes found in nature (natural photosynthesis) in the efficient transduction and conversion of solar energy. Hence, understanding the underlying mechanisms of energy transfer between NCs and anchored acceptor molecules is important to design novel nanohybrids that favor the singlet or triplet excited states using the perovskites as efficient photosensitizers to be applied in photocatalysts and light-harvesting applications.^5−7^

Singlet and triplet energy transfer, hereafter SET and TET, respectively, have been extensively reported in numerous interfacial processes in inorganic/organic systems.^8−15^ Although TET has been well demonstrated between perovskite nanocrystals (NC) and organic dyes in molecular hybrids,^16−26^ there are limited examples in the literature concerning the SET process,^27−34^ and even less employing ultrafast spectroscopy to fully unveil the exciton dynamics.^27,30−32^ The SET process can proceed via two mechanisms, i.e., Förster resonance ET (FRET) and Dexter ET (DET).^35,36^ The main difference between both mechanisms is the exponential dependence on the donor–acceptor distance. While FRET can operate over large distances (up to 10–12 nm) through dipole–dipole resonant coupling, DET is limited to shorter distances (up to 1–2 nm) and takes place via electron exchange.

Conversely, NC-sensitized TET has been developed as a promising route for triplet excited-state generation from organic dyes to molecular antenna complexes because of the unique properties of the NC sensitizer, such as wide absorption spectrum and large molar extinction coefficients, facile modulation of the photophysical properties, surface engineering, and small singlet/triplet state energy gap.^37−39^ The sensitization of these long-lived triplet excited states in organic molecules offers a wide range of applications in photodynamic therapy (PDT),^40,41^ redox photocatalysis,^42,43^ and photon upconversion.^44−46^ This TET mechanism, from the photoexcited NCs to surface-anchored organic molecules, can take place through the traditional simultaneous electron exchange (similar to DET),^16−20,22−24,26^ and the less frequent charge-transfer intermediate states.^20,21,25^ The presence of charge-separated states in hybrid materials and the subsequent charge recombination confirm the charge-mediated mechanism. In NCs, the rapid spin-flip phenomena of one of the carriers inside the nanocrystal, due to the strong spin–orbit coupling and symmetry-breaking phenomena, enables a high yield of molecular triplet generation.^25,47^

Borondipyrromethene (BODIPY) dyes have interesting photophysics that can be finely tuned for specific purposes.^48^ According to ring substitution of the BODIPY core, good emissive properties can be achieved. Functionalization with heavy atoms (bromine or iodine) or transition metals (Ir, Pt, Ru, Re, etc.) enables efficient intersystem crossing (isc) and populates the triplet excited state.^49^ Another strategy to generate the triplet excited state is by a sensitization process that combines charge separation (CS) and subsequent charge recombination (CR) or by triplet energy transfer from the sensitizer to the dye. The TET has only been reported for metal chalcogenide quantum dot nanohybrids (QD@BODIPY)^50,51^ and molecular dyads.^49,52^

In fact, we have recently demonstrated an efficient singlet sensitization of *meso*-(4-carboxyphenyl)BODIPY by CsPbBr_3_ NC, mediated by Förster resonance energy transfer (FRET, 85%), aided by the strong binding of the dye carboxylate group to the NC surface. The native oleate ligands were replaced by the dye carboxylic acid (X-type ligand), as was supported by Fourier transmission infrared (FTIR) and nuclear magnetic resonance (NMR) techniques.^15,53^ To the best of our knowledge, TET from a metal halide perovskite (donor) to BODIPY (acceptor) has not been reported yet.

Therefore, we investigate here the substantial role of the iodine substituent on the sensitization mechanism of two nanohybrids, which consists of the CsPbBr_3_ NC functionalized with two different BODIPY dyes bearing a carboxylic acid group as the anchoring group; specifically, 8-(4-carboxyphenyl)-1,3,5,7-tetramethyl-4,4-difluoro-4-bora-3a,4a-diaza-s-indacene (BDP) and 8-(4-carboxyphenyl)-2,6-diiodo-1,3,5,7-tetramethyl-4,4-difluoro-4-bora-3a,4a-diaza-s-indacene (I2-BDP).

A comprehensive study on the mechanism behind the energy/charge transfer between the NC and the dye in the two hybrids, under visible light, was performed by steady-state and time-resolved fluorescence spectroscopies, combined with an ultrafast technique, demonstrating an interesting control of FRET vs charge-mediated TET due to the iodine substituents in the BODIPY dye. Time-dependent density functional theory (TDDFT) quantum electronic calculation studies were also performed to predict the electronic properties of the dyes and support the ultrafast experimental findings. The mechanism of the singlet and triplet excited-state sensitization from NC to dyes, BDP and I2-BDP, was deeply analyzed. A charge-mediated TET mechanism that outcompetes the hot exciton thermalization and FRET processes in NC@I2-BDP led to the efficient generation of the I2-BDP triplet that can mediate the generation of singlet oxygen. As a proof of concept of the energy transfer photocatalytic activity of NC@I2-BDP, the oxidation of α-terpinene to ascaridole was evaluated. Thus, a tailored design of the hybrid nanomaterial properties is an appealing way to manipulate the excited-state dynamics of the nanohybrid, with a view to efficient visible-light harvesting to succeed in its use in photocatalysis.

It is noteworthy that our findings not only contribute to the fundamental understanding of the energy transfer mechanism between the CsPbBr_3_ NCs and BDP or I2-BDP dyes in the two novel nanohybrids but also evidence the dual role of the perovskite nanocrystals in NC@I2-BDP as efficient triplet photosensitizer and photocatalyst.

## Results and Discussion

### Preparation of Perovskite@BODIPY Nanohybrids

Highly colloidal CsPbBr_3_ NCs were first synthesized using the *hot-injection* approach with slight modifications (see the Supporting Information for further details and Scheme S1).^54^ Homogeneous cuboidal NCs with an average diameter of 6.4 ± 0.6 nm (Figure S1) were obtained, as confirmed by transmission electron microscopy (TEM) analysis. This size range is interesting because it is close to the Bohr exciton diameter (ca. 7 nm)^54^ and supports the energy transfer process.^34^

The BODIPY dyes were synthesized according to the reported procedures for 8-(4-carboxyphenyl)-1,3,5,7-tetramethyl-4,4-difluoro-4-bora-3a,4a-diaza-s-indacene (BDP),^55^ and 8-(4-carboxyphenyl)-2,6-diiodo-1,3,5,7-tetramethyl-4,4-difluoro-4-bora-3a,4a-diaza-s-indacene (I2-BDP),^56^ which were obtained with chemical yields of 13 and 80%, respectively (see the Supporting Information for synthetic details, Schemes S2 and S3, and Figures S2 and S3 for the ^1^H- and ^13^C NMR spectra of the purified products).

The presence of methyl groups directly anchored to the BODIPY core (particularly those at the 1,7-positions) in BDP caused a noticeable increase in the fluorescence quantum yield compared to *meso*-(4-carboxyphenyl)BODIPY^34^ due to free rotation blocking along the single bond between the phenyl and BODIPY moieties. The introduction of two iodine atoms appended to the 2,6-positions of the BODIPY core in I2-BDP was chosen to favor the intersystem crossing pathway via the heavy-atom effect, which can mediate the formation of the I2-BDP triplet excited state.

**Figure 1:**
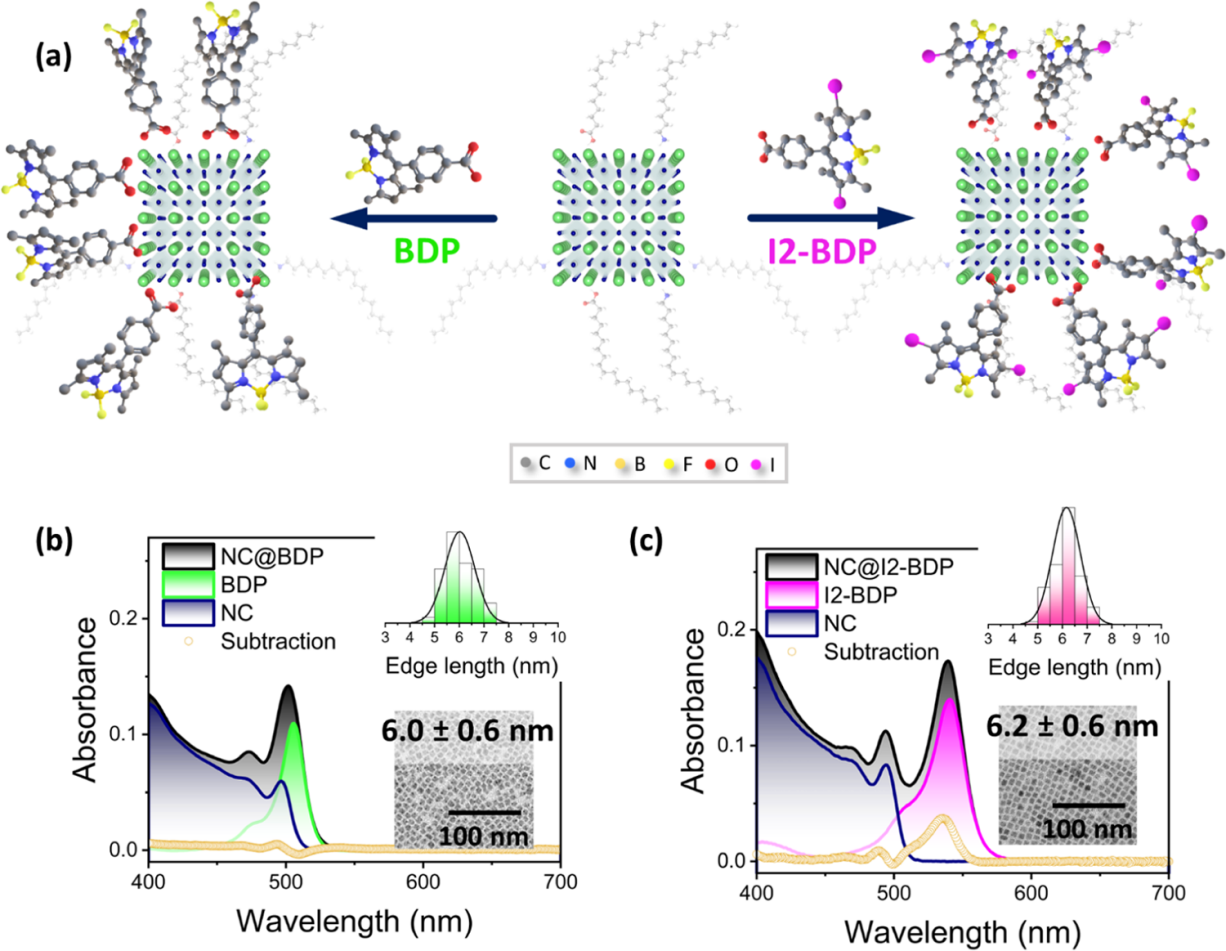
(a) Scheme of NCs before and after ligand exchange and the molecular structures of BODIPY dyes used to obtain NC@BDP and NC@I2-BDP nanohybrids. (b) Absorption spectra of NC@BDP, pristine NCs, and BDP in toluene. (c) Absorption spectra of NC@I2-BDP, pristine NCs, and I2-BDP in toluene. The subtraction spectrum is shown (orange). Both insets show histograms accounting for 100 nanocrystals from TEM images. The scale bar is 100 nm.

Nanohybrid preparation was carried out by a ligand-exchange procedure (see Figure 1a and Supporting Information for further details).^21^ Briefly, an excess of BODIPY powder was added to a colloidal dispersion of CsPbBr_3_ NCs capped with oleic acid and oleylamine in hexane and stirred for 15 min. Owing to the low solubility of BODIPYs in hexane, the mixture was filtered. Then, the filtrate, containing the corresponding nanohybrid, was collected for subsequent analysis.

### Optical and Electronic Properties of Perovskite@BODIPY Nanohybrids

The optical properties of NC@BODIPY nanohybrids in toluene (Figure 1b,c) were compared with the absorption spectra of NCs and BODIPY dyes, prepared under the same conditions as those of the corresponding nanohybrids (OD = 0.1).

The NCs showed an excitonic peak centered at 496 nm, which is in a weak quantum confinement regime, and corresponds to a 6.4 ± 0.6 nm nanocrystal size, as confirmed by transmission electron microscopy (Figure S1).^2,57^ It is noteworthy that after the ligand exchange, the size and morphology of the NCs were preserved, with values of 6.0 ± 0.6 and 6.2 ± 0.6 nm for NC@BDP and NC@I2-BDP nanohybrids, respectively (inset, Figure 1b,c). The BDP absorption maximum (506 nm) was close to that of the NC exciton, as shown in Figure 1b, in contrast to that of I2-BDP, with an absorption maximum at 540 nm, as shown in Figure 1c. Thus, the iodine substituents of I2-BDP produced a significant effect on the absorption properties of NC@I2-BDP,^58,59^ namely, a redshift of the peak at ca. 50 nm, thus improving the dye light absorption in the visible window. The contribution of BODIPY dye absorption was observed in both nanohybrids. Based on the molar extinction coefficients of CsPbBr_3_ NCs (3.94 × 10^6^ M^–1^ cm^–1^ at 450 nm), BDP (5.34 × 10^4^ M^–1^ cm^–1^ at 506 nm), and I2-BDP (2.58 × 10^4^ M^–1^ cm^–1^ at 540 nm), dye/NC molar ratios of 80 and 250 were calculated for NC@BDP and NC@I2-BDP, respectively, thus suggesting a better interaction of I2-BDP with the NC surface.^26^ The small shift in the I2-BDP dye in the NC@I2-BDP nanohybrid absorption spectrum could be assigned to solvation or surface crowding of the dye. Note that the subtraction spectra of the contribution of the dye and the NC to the NC@I2-BDP spectrum (maximum ca. 544 nm, Figure 1c) were only observed for NC@I2-BDP (dye/NC 250), which could be assigned to the electronic coupling between I2-BDP dye molecules on the NC surface due to their ability to form π-staking dimers, aggregates, and self-assemblies.^60−62^

**Figure 2:**
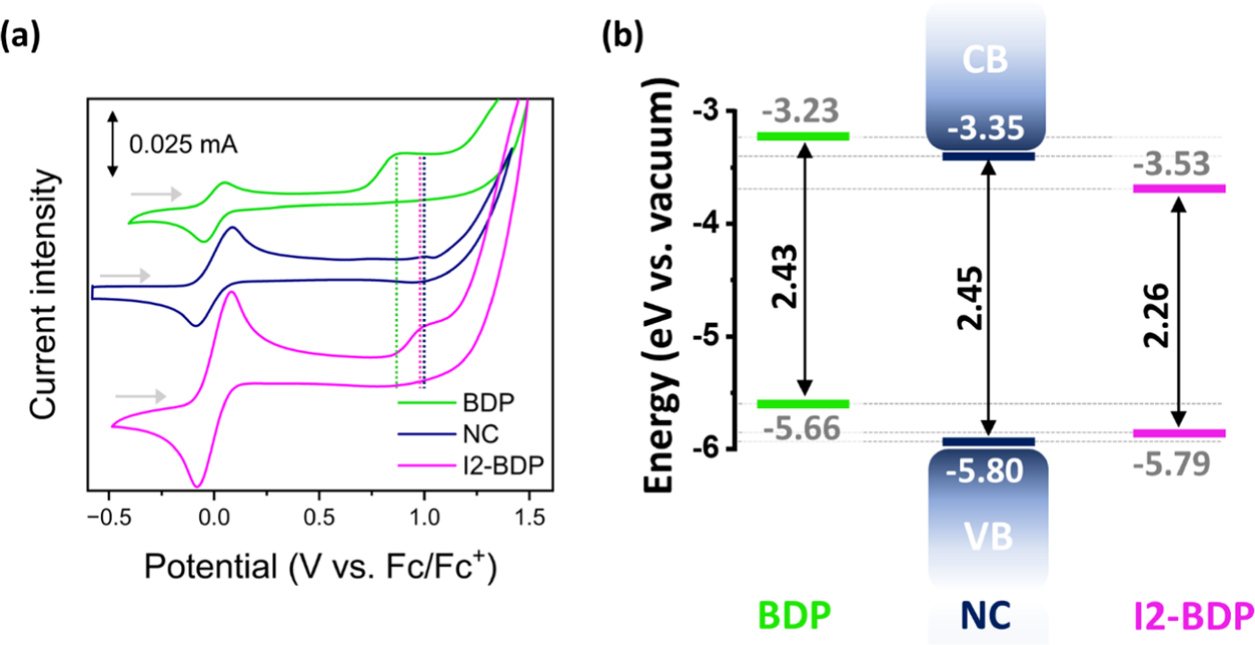
(a) Cyclic voltammograms vs the Fc/Fc^+^ redox couple for the anodic regions of NC and BODIPY dyes in a mixture of acetonitrile (ACN)/toluene (1:3 v/v). The dashed lines represent the irreversible redox peaks. The scan rate is 0.2 V s^–1^. Arrows indicate scan directions. (b) Calculated energy levels for the CsPbBr_3_ NC (blue), BDP (green), and I2-BDP (pink).

Electrochemical measurements were carried out since cyclic voltammetry (CV) can provide valuable information about the energy levels of the valence band (VB) and conduction band (CB) of the semiconductor nanocrystal and the frontier molecular orbitals (the highest occupied molecular orbital (HOMO) and the lowest unoccupied molecular orbital (LUMO)) of the dye.^63^ The cyclic voltammograms in the anodic and cathodic directions are illustrated in Figures 2a and S4, respectively, and the data are summarized in Table S1. Both BDP and I2-BDP showed irreversible anodic waves (*V*_ox_ peak values of +0.86 V vs Fc/Fc^+^ and +0.99 V vs Fc/Fc^+^, respectively), while the NC produced a peak value of +1.00 V when scanning forward at 0.2 V s^–1^.

Scanning in the cathodic direction showed irreversible waves (*V*_red_ peak values of −1.63 V vs Fc/Fc^+^ and −1.58 V vs Fc/Fc^+^ for BDP and I2-BDP, respectively) and a peak value of −1.53 V vs Fc/Fc^+^ for the NCs. Determining the redox events in NCs is somewhat challenging owing to low material stability^64^ and the high dependence of the energy levels on their composition, size, shape, and surface chemistry.^65,66^

The calculated electrochemical gaps (Δ*E*^CV^) were 2.49, 2.57, and 2.53 eV for BDP, I2-BDP, and NCs, respectively. These values are quite similar to those obtained from the optical data for BDP and NCs (Figure S5), whereas they deviate a bit for I2-BDP. The singlet-state energies^67,68^ of BDP and I2-BDP were found to be 2.43 and 2.26 eV, respectively, obtained from the crossing point between the normalized absorption and fluorescence spectra; while the Tauc plot method^69^ was used to calculate the optical band gap (Δ*E*^Opt^ = 2.45 eV) for the NC, in agreement with the reported values in the literature.^65,70−72^ An acceptable approach to estimate the LUMO/CB energy is to perform a subtraction between the HOMO/VB and S_1_/Δ*E*^Opt^ obtained through electrochemical and spectroscopic techniques, respectively. This methodology is widely used for perovskites due to the low stability of this material toward cathodic potentials.^73^

Based on the relative position of the energy levels depicted in Figure 2b, energy transfer (ET) from the NC to the corresponding dye was expected because it is thermodynamically downhill in energy. In addition, charge transfer events from the NC to the dyes should also be considered. The estimated driving forces (−Δ*G*) of 0.18 eV for electron transfer (eT) and 0.01 eV for hole transfer (hT) from the NC to I2-BDP indicate that these processes are thermodynamically favorable, while hT is only expected for BDP with a −Δ*G* of 0.14 eV, whereas eT is not allowed since the LUMO level is 0.12 eV higher than the CB of NCs.

### Excited-State Interactions in NC@BODIPY Nanohybrids and Time-Resolved Analysis

**Figure 3:**
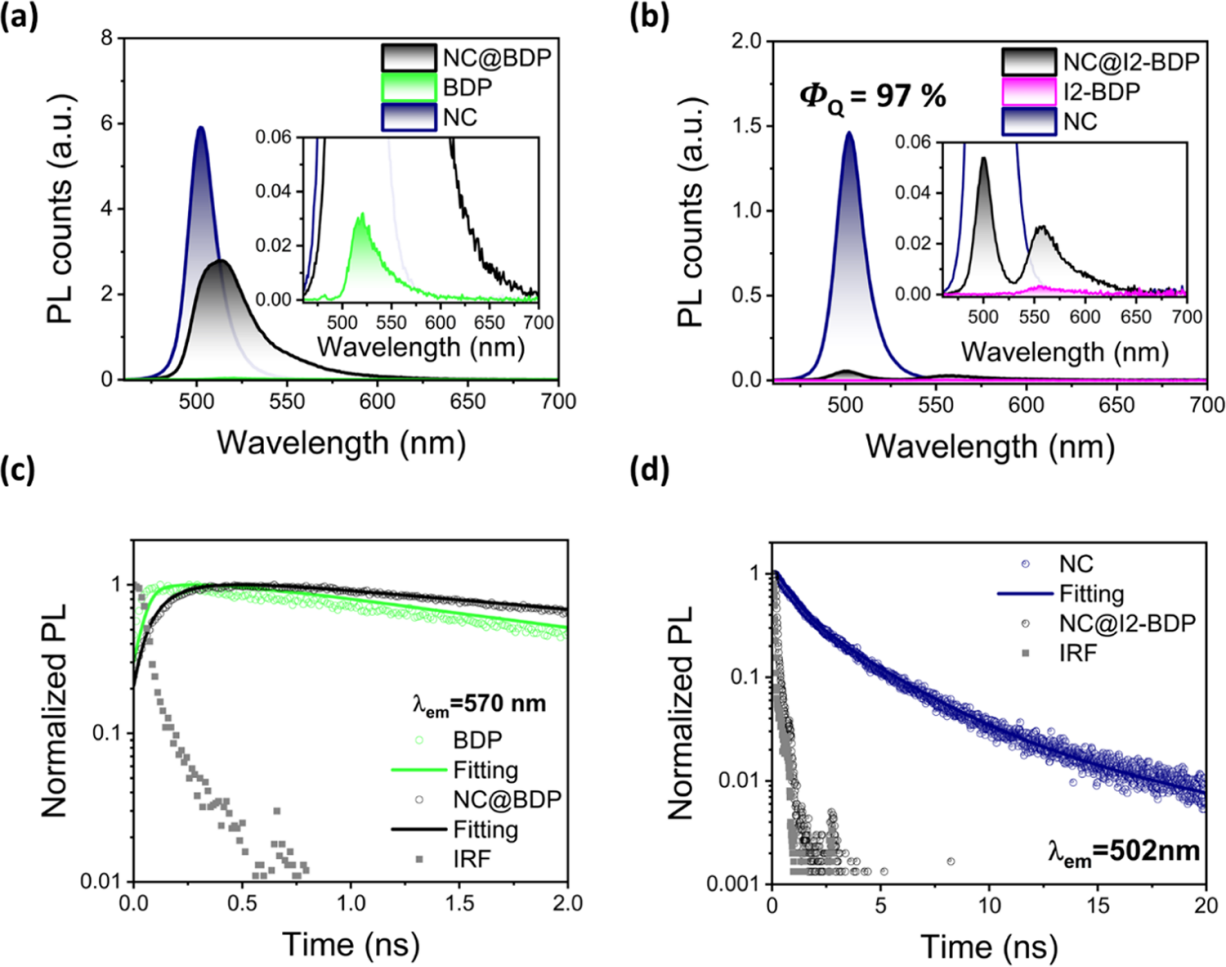
Steady-state and time-resolved optical properties. PL spectra of (a) NC@BDP and (b) NC@I2-BDP nanohybrids along with their respective controls at the same conditions in toluene at 420 and 450 nm excitation wavelengths, respectively. Both insets show enlarged emission windows. (c) PL decay traces were monitored at 570 nm, where only BDP was emissive. (d) PL decay traces were monitored at 502 nm, where only NCs were emissive. Pulsed laser excitations of 420 and 450 nm (9.8 MHz) were used for (c) and (d), respectively. The instrument response function (IRF) was ca. 150 ps, calculated at full width at half-maximum (fwhm).

The photoluminescence (PL) of the NC in NC@BDP upon photoexcitation at 420 nm (where the NC mainly absorbs) was completely quenched compared to that of the pristine NC, which presented a moderate PL quantum yield (Φ_PL_) of 33 ± 1% (Figure 3a). However, the emission of BDP in NC@BDP at 600 nm, where there was no NC PL contribution, was found to be 50-fold higher than that of the BDP sample prepared at the same conditions (inset Figure 3a). These data suggest that the BDP emission results from a photosensitization process.

In the case of NC@I2-BDP, an excitation wavelength of 450 nm was used to selectively photoexcite the NC in the nanohybrid (Figure 3b), owing to the different absorption features of I2-BDP.^74^ Although a quenching efficiency Φ_Q_ of 97 ± 1% was calculated, low sensitized emission of I2-BDP in NC@I2-BDP was observed (9-fold higher emission than that of I2-BDP control), which may be due to the poor fluorescence quantum yield (Φ_F_) of the I2-BDP dye (2.7 ± 0.3%, similar to the reported data),^75^ or to the weak energy transfer efficiency (Φ_ET_) between the NC and I2-BDP dye.

Time-resolved PL was employed to study the exciton recombination dynamics from the band edge using a time-correlated single photon counting (TCSPC) technique. The pristine NC PL decay trace (excited at 420 nm and recorded at 502 nm, Figure S6) was fitted to triexponential decay functions τ_1_ = 0.81 ± 0.01 ns, τ_2_ = 3.42 ± 0.02 ns, and τ_3_ = 11.71 ± 0.12 ns, which corresponds to surface nonradiative, direct electron–hole radiative, and trap-assisted recombination mainly for holes, respectively.^76−80^ The shortest component was significantly quenched (81 ± 3%) with a time constant of 154 ± 2 ps in NC@BDP, whereas the other components were less affected (20–40%, see Table S2 for fitting parameters and details for the fitting model in Supporting Information).^81^

**Figure 4:**
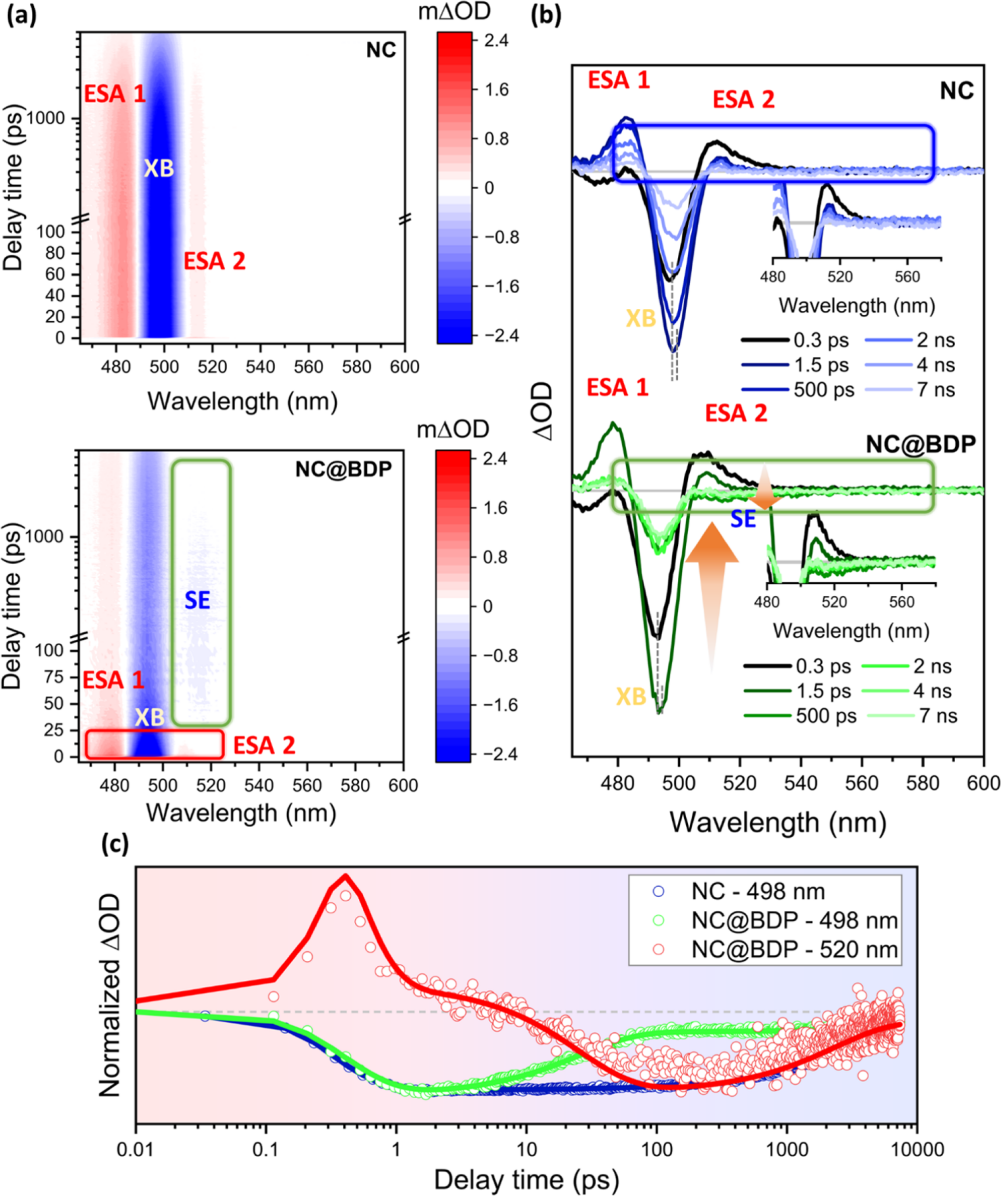
Time-resolved transient absorption (TA) data of pristine NCs and NC@BDP nanohybrids in toluene using a 420 nm pump. (a) Two-dimensional (2D) pseudocolor TA plots of NCs (top) and NC@BDP (bottom). (b) TA spectra at indicated delay times. Inset: enlarged TA region. (c) Kinetics of pristine NCs and NC@BDP nanohybrids (hollow circle) probed at wavelengths of 498 and 520 nm and the best multiexponential fittings (solid line). The kinetics are normalized for better comparison.

The fluorescence decay of BDP at 570 nm in NC@BDP (where the contribution of the NC PL emission is negligible) was fitted to a multiexponential model with an evidently increased component (τ_rise_) of ca. 110 ps, which was absent in the BDP trace (Figure 3c). Although this observation seems to agree with the sensitization process by the NC, this lifetime is close to the resolution limit of the TCSPC setup (ca. 150 ps at fwhm). In the case of NC@I2-BDP, the PL decay recorded at 502 nm was completely quenched below the TCSPC setup resolution (Figure 3d), while the PL trace decay, monitored at 600 nm, where only I2-BDP emits, presents an almost identical trace to that of I2-BDP (Figure S7). Selective excitation of the dye in both nanohybrids (above 500 nm) resulted in decay traces similar to those of the pristine dyes (Figure S8). Limitations in TCSPC setups can hinder a deep analysis of PL lifetimes. In order to investigate the dynamics of hot- and band-edge excitons and the evolution of charge carriers,^82,83^ femtosecond transient absorption spectroscopy (fs-TAS) was performed with a time resolution of 250 fs (see Supporting Information for the pump–probe experimental details). Two-dimensional pseudocolor spectra of NCs and NC@BDP nanohybrids as a function of the probe wavelength and delay time are shown in Figure 4a, following 420 nm laser pulse excitation (3.0 eV). After excitation of NCs, there are several signals: (i) a strong exciton bleaching (XB) feature at 498 nm, assigned to state-filling induced bleaching; (ii) the excited-state absorption at 485 nm (ESA 1) ascribed to energy transitions of the exciton absorption; and (iii) the excited-state absorption at 510 nm (ESA 2) related to vibrational cooling to the band edge.^84,85^ The spectral evolution in NCs at early times (0.3 ps) showed a redshift and a broadening of the XB, which, along with the disappearance of ESA 2, confirms the presence of hot carrier relaxation in the nanocrystal (produced by the above band-edge pump pulse), whereas the presence of ESA 1 arises from the population of the band edge.^86−88^ It has been described that the electron and hole levels contribute to ca. 67 and 33% to the XB feature, respectively, while in the case of the traditional metal chalcogenide quantum dots, the electrons present the main contribution.^86^ Thus, exciton dissociation and extraction and trapping of one of the charge carriers will affect the XB feature amplitude. The subsequent charge recombination (CR) process can be probed, as the XB feature still contains the contributions of one of the leftover charge carriers.^86,89^

The XB recovery kinetics at 498 nm were evaluated to obtain quantitative information on the exciton/charge carrier dynamics at the NC and nanohybrid interfaces. Pristine NC kinetics was fitted to a multiexponential function (see Supporting Information for fs-TA fitting details, eq S7); the obtained time constants were 0.28 ± 0.04 ps (rise), 151 ± 52 ps (5%), and 3.57 ± 0.09 ns (95%) (Table S4), which could be ascribed to vibrational cooling, surface state related-charge trapping,^90^ and electron–hole direct radiative recombination, respectively. An offset was necessary to account for the longest component. The electron–hole radiative recombination is the dominant pathway in the pristine NCs under these conditions, suggesting a minor contribution of hole trapping to the overall decay.

Strong quenching of the XB and ESA 1 features (Figure 4a, bottom), indicative of exciton dissociation due to the creation of a new nonradiative pathway, was observed in NC@BDP with the concomitant observation of a negative signal at 520 nm assigned to the stimulated emission (SE) of the BDP dye (see Figure S9 and Table S5). Figure 4b shows the TA spectra at the indicated delay times for pristine NCs and NC@BDP. The SE band appears in the 1.5–500 ps time range together with the quenching of the XB feature, denoting an SET process.

The normalized kinetic traces are shown in Figure 4c. The best multiexponential fitting of the XB kinetic traces showed three components: 0.29 ± 0.07 ps (rise), 26 ± 1 ps (85%), and 7 ns (15%). The longest was fixed according to the data obtained from TCSPC. Strong quenching of the electron–hole radiative recombination together with a short component at 26 ps confirms the SET from NC to BDP as the main process, with a rate constant of 3.8 ± 0.2 × 10^10^ s^–1^. This process is of the same order as the diffusion rate constant (≈10^10^ s^–1^),^91^ indicating that BDP is strongly anchored to the NC surface, as confirmed by FTIR (see Figure S10 and the discussion in SI). The absence of the stretching bands corresponding to carboxylic acid at 1680 cm^–1^ (BDP) and 1716 cm^–1^ (I2-BDP) in the nanohybrid confirmed the binding of the dyes to the NC surface by the −COO^–^ groups and the absence of free dyes in the solution. These findings are consistent with those in previous works.^34,92,93^

**Figure 5:**
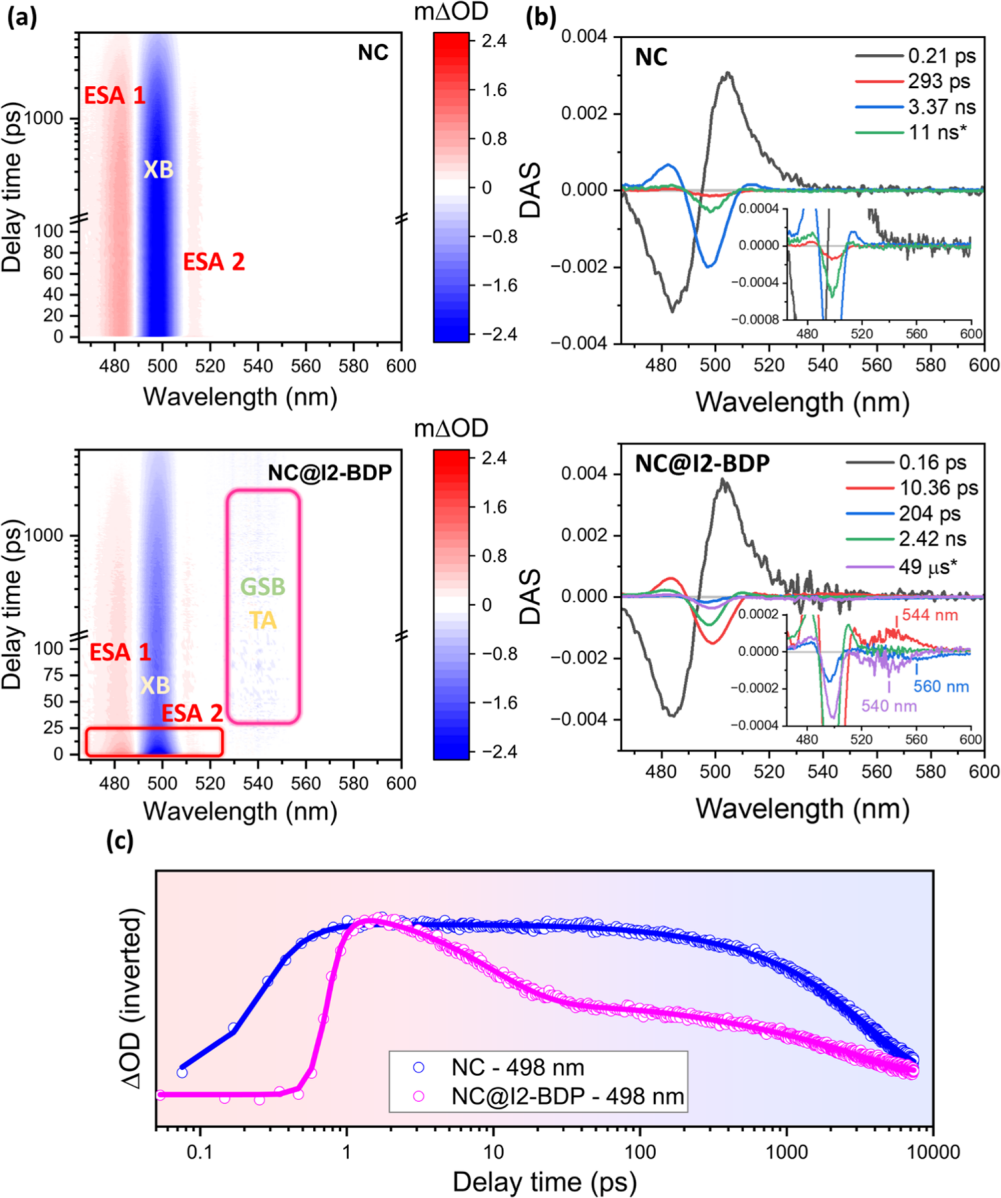
Time-resolved TA data of the pristine NC and NC@I2-BDP nanohybrid in toluene with a 450 nm pump. (a) 2D pseudocolor TA plots of NCs (top) and NC@I2-BDP (bottom). (b) Decay-associated spectra (DAS) obtained using GA for NC (top) and NC@I2-BDP (bottom) under 450 nm pump excitation. (c) Inverted kinetics of pristine NCs and NC@I2-BDP nanohybrid (hollow circle) probed at 498 nm, and the best multiexponential fittings (solid line).

In the case of NC@I2-BDP, according to the energy levels obtained from the electrochemical and spectroscopic analyses (Figure 2b), charge transfer pathways (electron/hole transfer) are also feasible. Figure 5 shows the fs-TA results for NC@I2-BDP. Although the photoexcitation of the pristine NC at 450 nm gave rise to the same components as the NC excited at 420 nm (XB and both ESA features, see Table S4 for the fitting parameters), a shortening of the carrier cooling time from 0.28 to 0.22 ps was observed, in agreement with the reported dependence of this process with the excitation wavelength.^88^ XB quenching of the NC in the NC@I2-BDP was faster than that in NC@BDP as can be seen in Figure 5a, along with the observation of a low intense and noisy band between 520 and 560 nm, where the GSB feature of the I2-BDP was expected (Figure S9 and Table S5). Kinetic trace analysis (Figure 5c) showed the following time constants: 0.18 ± 0.08 ps (rise), 9.5 ± 0.4 ps (55%), 273 ± 8 ps (11%), and 2.8 ± 0.2 ns (34%). The charge transfer of one of the hot charge carriers from the NC to the surface-anchored I2-BDP dye can be attributed to the subpicosecond component, owing to the strong electronic coupling of the wave functions between the NC and I2-BDP dye, as confirmed by the shift in Figure 1c, which outcompetes vibrational cooling.^94^ This is a very interesting observation since the thermalization of hot carriers typically constitutes a major energy loss channel, responsible for the Shockley–Queisser efficiency that limits their photovoltaic application.^95^ Thus, considering the aforementioned findings and that hot holes can be more easily extracted than electrons in hybrid perovskites,^96^ an ultrafast hot hole transfer with a rate constant of ca. 1.00 ± 0.08 × 10^12^ s^–1^ from the NC to I2-BDP can be established. Similar values have been observed for other hot charge carrier transfers from perovskite NCs to 4-mercaptophenol, benzoquinone, and phenothiazine.^94,97^

The TA spectra at the indicated delay times (0.3 ps–7 ns) are shown in Figure S12a. Kinetic traces recorded at 560 nm (Figure S12b) were assigned to the SE of the dye (Figure S9) probing the population of the singlet excited state (^1^I2-BDP*), which takes ca. 20–30 ps to reach the maximum intensity. This finding occurs in the same order as the approximately 10 ps component extracted from the XB feature analysis (pink circles). This could indicate some correlation between the two processes (see the discussion below).

To get a deeper insight into the component analysis, global analysis in GloTarAn software (GA, details shown in Supporting Information) across the entire spectrum was used to extract more information on the spectral data, combining information from multiple time-delayed spectra, and the corresponding decay associated spectra (DAS), as illustrated in Figures 5b and S11 (see Table S6 for fitting data). A singular value decomposition (SVD) procedure was applied to the three-dimensional (3D) data matrix, and components similar to those extracted by single wavelength analysis were obtained for pristine NCs from GA. The amplitude of DAS represents the contribution of that component to the fitting. A similar spectral shape has been reported through GA in CsPbBr_3_ NCs.^88^ It is noteworthy that a 11 ns time constant was fixed based on the last component obtained by TCSPC analysis. The GA results for NC@BDP showed the same number of components as those obtained by single wavelength analysis. A time constant of ca. 26 ps (Figure S11) perfectly matches the XB component of the NCs (Figure 4c and Table S4), whereas the component of ca. 8 ns exhibits spectral features of both NCs and SE of BDP (515 nm), which are similar to those obtained from the sensitization process of BDP, as seen from TCSPC (Table S2). The last component of 5 μs was fixed in the analysis, which is explained later. No triplet excited state of BDP was detected (Figures S9 and S13). A similar analysis was performed for NC@I2-BDP, where the hot hole transfer, on a subpicosecond time scale, was previously described. Interestingly, a new component with ca. 10 ps with an absorption band at around 544 nm (Figure 5b, bottom) was detected together with the XB feature, which was attributed to I2-BDP^+•^ species, thereby supporting charge separation in the previous stage and subsequently leading to charge recombination in ca. 10 ps. The spectral features at 204 ps showed two main components, XB and I2-BDP SE (560 nm), which were ascribed to the lifetime of the sensitized emission of I2-BDP. The short fluorescence lifetime of the I2-BDP dye (see Figure S8 and Table S3) is expected, as the intersystem crossing (isc) due to the heavy-atom effect gives rise to a favored triplet excited state (Figures S9 and S13). Therefore, the ET process seems to occur at an early stage. The component obtained at 2.42 ns only describes the NC fraction that eventually does not recombine to yield ^1^I2-BDP* since only the XB feature was detected, while the 49 μs component was fixed during the analysis due to the population of the triplet state. This lifetime obtained from ns-TA measurements for the I2-BDP dye (Figure S13) corresponds to the excited triplet state of the I2-BDP dye, as was reported elsewhere.^98^

It has been reported that CR in nanohybrids could lead to triplet state formation due to the low-defined spin configuration and rapid spin-flip of the carrier in nanocrystals.^25,90,99^ Therefore, based on the contributions of the NC XB and I2-BDP GSB features associated with the 49 μs component (Figure 5b bottom), the population of the I2-BDP triplet state through CR as an additional pathway can be proposed.^50,90^ The NC XB contribution to this triplet excited-state component has already been observed in other works and it has been attributed to the Stark effect induced by the electric fields of surface-bound ^3^I2-BDP* and charge distribution within the NCs.^25,100,101^ All kinetic parameters obtained from GA are collected in Table S6. The most relevant finding from the fs-TA analysis of the nanohybrids can be summarized as (i) the observation of the SE of the dyes; (ii) a remarkable quenching of the NC XB feature; (iii) a hot hole transfer in a subpicosecond time scale, which suggests the formation of I2-BDP^+•^ species; and (iv) the observation of the sensitized triplet excited state of I2-BDP.

With the aim of confirming the nature of the species generated by the charge transfer from NC to I2-BDP, as observed by fs-TA spectroscopy analysis, some spectroelectrochemical measurements were performed. Figure S14 shows the UV–vis absorption spectra recorded before and after applying a bias, as well as the difference spectra of I2-BDP for cathodic and anionic potentials that correspond to I2-BDP^+•^ and I2-BDP^–•^. The band observed below 560 nm when the anodic potential was applied can be identified as I2-BDP^+•^ and agrees with the positive band observed at ca. 544 nm in the decay-associated spectra in Figure 5b, bottom.

### Electronic Quantum Chemical Calculations

Theoretical calculations were carried out using density functional theory (DFT) and time-density functional theory (TDDFT) to understand the photophysical properties of the BDP and I2-BDP dyes. DFT is commonly used in quantum mechanics to calculate electronic structures, while TDDFT can predict excited-state properties and electronic transitions. The computed ground-state equilibrium structures of BDP and I2-BDP derivatives are shown in Figure S15. The calculations indicate an almost planar structure for the BODIPY core and almost an orthogonal orientation of the phenyl-COOH moiety in BDP. For I2-BDP, slight deviations in planarity or orthogonality compared with BDP are suggested.

TDDFT was used to assign the first singlet excitation (π–π* transition) as that resulting from the highest occupied molecular orbital (HOMO) to the lowest unoccupied molecular orbital (LUMO).^102^ In Figure S16, the HOMO and LUMO of BDP are localized on the BODIPY core, while for I2-BDP, the electron density was also computed for the two iodine atoms (HOMO orbital). Due to the deviation from orthogonality, the MOs calculated for the optimized geometries display some minor density contribution to the phenyl-COOH substituents, especially for I2-BDP (LUMO orbital).

As expected, the S_1_ state is dominated by H → L excitation (Table S7). The molecular orbitals involved in the transitions also reveal the charge transfer character for the 486 nm for BDP and 499 nm for the I2-BDP transitions, arising from the shift of the electron density from the phenyl-COOH ring to the BODIPY core upon excitation (Figure S17). The optimized geometries and natural transition orbitals (NTOs) of I2-BDP in three different forms (neutral, radical anion, and radical cation) were also determined (Figures S18 and S19). The predicted absorption spectra and the spin density surfaces of the radical anion and the radical cation of I2-BDP were successfully obtained by TDDFT. The spin density of the radical anion is entirely restricted to the BODIPY moiety, while the spin density surface of the radical cation is distributed between the BODIPY core and the phenyl-COOH group.

The calculated absorption spectra, HOMO and LUMO energies of I2-BDP (neutral, radical cation, and radical anion), and the oscillator strengths from TDDFT are shown in Figure S19. The neutral form has absorptions at ca. 469 and 372 nm (see Table S8 and Figure S16), which are shifted to 488 and 347 nm in the radical cation form. Moreover, an additional peak at a longer wavelength (ca. 572 nm) was detected with an oscillator strength of 0.092 (Figure S19). In the radical anion form, only two peaks were observed at 459 and 369 nm.

Based on the fs-TA spectral data and the predicted excited state from TDDFT electronic quantum calculations (Table S8), the following picture for I2-BDP can be highlighted: (i) a transient band at ca. 544 nm in toluene (Figure 5b, bottom); (ii) an S_1_ → S_11_ transition at 572 nm (*f* = 0.001) for the neutral form; (iii) an S_1_ → S_8_ transition at 574 nm (*f* = 0) for the radical anion; and (iv) an S_1_ → S_11_ transition at 576 nm (*f* = 0.144) for the radical cation. The theoretical predictions (wavelength maxima and oscillator strength, *f*) agree with the experimental data and support the formation of I2-BDP^+•^ as an intermediate in the sensitization of I2-BDP in the NC@I2-BDP nanohybrid.

### Proposed Sensitization Mechanism in the Nanohybrids

**Figure 6:**
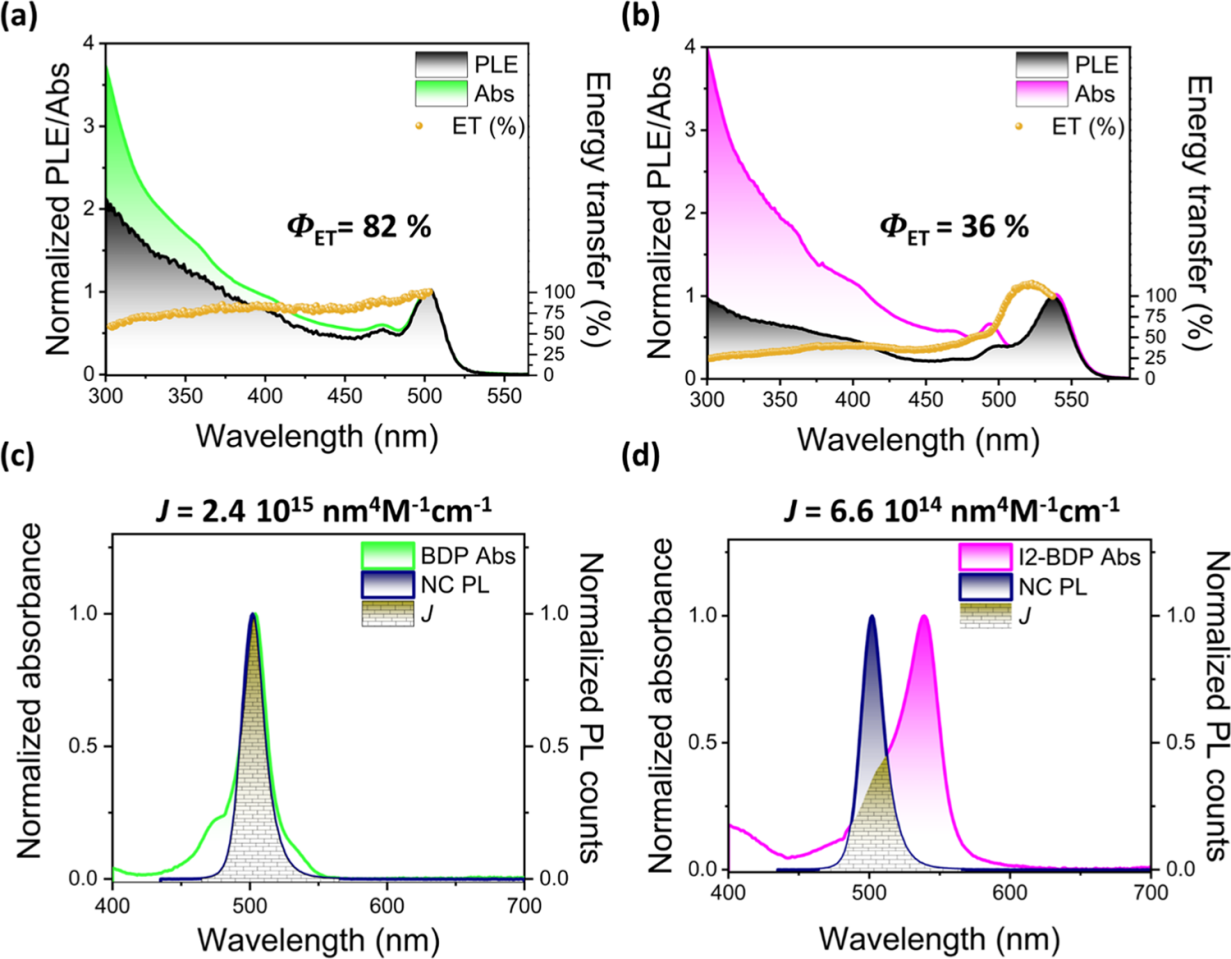
Steady-state optical properties. Comparison of PLE and absorption spectra and their corresponding ratios to estimate energy transfer efficiency (Φ_ET_) for (a) NC@BDP and (b) NC@I2-BDP nanohybrids in toluene. The PLE spectra were monitored at emission wavelengths of 570 and 600 nm for (a) and (b), respectively. Representation of the spectral overlap (*J*) between the NC PL and (c) BDP and (d) I2-BDP absorption at 420 and 450 nm excitation, respectively.

Although a high PL quenching efficiency, determined by time-resolved PL and fs-TA analysis, was observed for both nanohybrids, the iodine substituent in the BODIPY core played a key role in the sensitized energy transfer mechanism between the NC and the anchored dye. Figure 6a,b displays a comparison between the photoluminescence excitation (PLE) and absorption spectra, both normalized to the dye absorption band maximum for NC@BDP and NC@I2-BDP, respectively. It is widely known that the PLE spectrum recorded at the emission wavelength probes the origin of a given emission. Therefore, the presence of NC absorption features in the PLE spectra is irrefutable evidence of sensitized emission of the dye by the NC in the corresponding nanohybrid (Figure S20). To estimate the contribution of ET in each system, both spectra were normalized to the absorption maximum of the acceptor, i.e., 506 and 540 nm for BDP and I2-BDP, respectively. Thus, the magnitude of the ratio between the PLE and absorption spectra (at excitation wavelength maxima of 420 and 450 nm for NC@BDP and NC@I2-BDP, respectively) allows the estimation of the ET contribution from the selectively photoexcited NC (donor) to the surface-anchored BODIPY dye (acceptor).^34,103^ The significantly higher Φ_ET_ (ca. 82%) obtained for NC@BDP compared to NC@I2-BDP (ca. 36%) is consistent with the greater spectral overlap (*J*) between the donor emission and acceptor absorption (Figure 6c,d). Interestingly, the contribution of the ca. 26 ps component to the overall XB feature in the fs-TA studies was 85%, which agrees with the PLE studies from steady-state fluorescence spectroscopy.

Considering the noticeable spectral overlap between the NC emission and the BODIPY absorption (*J* = 2.4 × 10^15^ and 6.6 × 10^14^ nm^4^ M^–1^ cm^–1^ for BDP and I2-BDP, respectively) in Figure 6c,d, the FRET mechanism is expected, as has been reported for the energy transfer from CsPbBr_3_ nanocrystals to rhodamine B (*J* > 2.04 × 10^14^ nm^4^ M^–1^ cm^–1^).^30^ The FRET efficiency, hereafter Φ_FRET_, can be expressed as indicated in eq 1:
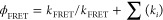
1where *k*_FRET_ stands for the Förster resonance ET rate constant and ∑(*k*_*i*_) for all the remaining processes that deactivate the NC excited state (radiative and nonradiative processes). Assuming only FRET as the depopulation via the NC excited state, the calculated Φ_FRET_ values are 98 and 94% for NC@BDP and NC@I2-BDP, respectively, higher than the PLE experimental analysis (see Supporting Information for calculation details).

The calculated *k*_FRET_ in NC@BDP is 1.51 × 10^10^ s^–1^, which is of the same order as the experimental constant determined by fs-TA kinetics (3.8 × 10^10^ s^–1^) and TCSPC (9.09 × 10^9^ s^–1^) technique. However, for NC@I2-BDP, the difference is higher, indicating a predominant contribution of another nonradiative pathway in the NC excited state, such as charge transfer as was discussed above.

To identify the sensitized triplet excited-state formation of the dyes, singlet oxygen (^1^O_2_) phosphorescence measurements were performed at 1275 nm for the nanohybrids.

**Figure 7:**
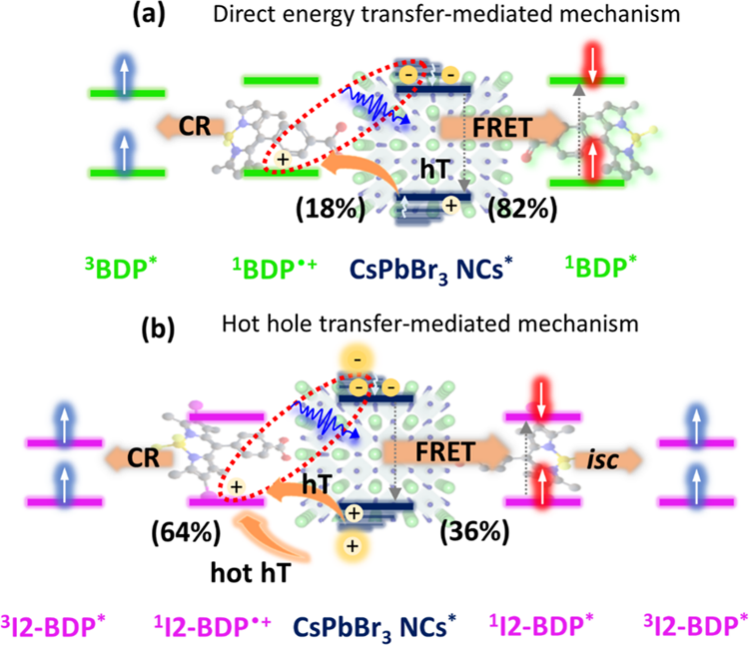
Schematic diagrams of the proposed sensitization mechanisms for (a) BDP and (b) I2-BDP dyes in NC@BODIPY nanohybrids. The OA and OAm ligands are not shown for clarity.

The ability to generate singlet oxygen suggests that the excited triplet states are accessible and may play a significant role in the photochemistry and photophysics of the nanohybrids. In general, BODIPYs do not undergo efficient isc to the triplet excited state,^58^ but BODIPYs functionalized with heavy atoms (such as bromine or iodine) can increase the *isc* quantum yield (Φ_isc_) due to the enhancement of spin–orbit coupling, and can act as photosensitizers of singlet oxygen (^1^O_2_) in PDT and also in triplet–triplet annihilation upconversion and redox photocatalysis.^49,104,105^ The position of the halide atom connected to BODIPY has a remarkable impact on the photosensitizing properties, particularly when it is directly appended to the BODIPY core.^58^ As expected, the nanohybrids presented a different response to ^1^O_2_ generation (Figure S21). Optically matched solutions of I2-BDP and NC@I2-BDP dispersions at 550 and 450 nm, respectively, yielded the same intensity of ^1^O_2_ phosphorescence emission, supporting the efficient I2-BDP triplet state population sensitized by the NCs. Control experiments were conducted to rule out the possibility of sensitization by the pristine NCs and I2-BDP at 450 nm. Surprisingly, NC@BDP yielded a small but detectable amount of ^1^O_2_ through selective excitation of NC at 420 nm. Thus, selective photoexcitation of NCs yielded a small contribution from the triplet state in the BDP dyes, which was inaccessible via direct excitation of BDP at 515 nm (Figure S21). We believe that this triplet sensitization in NC@BDP takes place because of a residual charge-mediated pathway, as occurred in NC@I2-BDP. Nevertheless, the contribution of this charge-mediated pathway should be small. As presented earlier, hole transfer from NC to BDP could also be a plausible mechanism for deactivating the NC excited state. The CR of this charge-separated state (CSS) could generate the triplet excited state in BDP dyes (^3^I2-BDP*) as it has been reported in other systems.^21,25^ According to the previous analysis, we propose that the main mechanism contributing to BODIPY sensitization is FRET for NC@BDP, and a hole charge transfer mechanism for NC@I2-BDP (Figure 7).

As depicted in Figure 7a, FRET is the major contributor (82%) to BDP sensitization in NC@BDP (right pathway), as confirmed by steady-state and fs-TA spectroscopy studies. The relatively small proportion of ^1^O_2_ phosphorescence detected in NC@BDP under selective photoexcitation of NC can be understood if the quenching of the remaining NC excited state is attributed to a hole transfer process from the NC VB to the BDP HOMO (18%), followed by charge recombination to produce the triplet (left pathway), considering the energy level alignment shown in Figure 2b. The accessibility of BDP triplets through sensitization by NC via hole transfer and subsequent charge recombination was addressed, while BDP dyes *per se* did not generate molecular triplets due to inefficient intersystem crossing.

In the case of NC@I2-BDP, a rather different picture is obtained for the sensitization of I2-BDP; indeed, in this case, hot hole carriers (ca. 21%) were quickly transferred from the NC VB to the I2-BDP HOMO resulting in a CSS (64%). This CSS is formed by hole transfer from vibrationally hot and relaxed states. Given the low energy of the I2-BDP triplet state (1.62 eV, see Figure S22)^106^ with respect to that of the CSS in toluene and the ill-defined spin configuration in NC, due to the strong spin–orbit coupling and state mixing,^25^ the subsequent CR process led directly to a molecular triplet in I2-BDP (left pathway), as evidenced by the long-lived XB and GSB of I2-BDP of 49 μs (Figure 5b, bottom). However, the remaining contribution for I2-BDP sensitization (36%) stems from FRET to yield a molecular singlet in I2-BDP (right pathway), as we confirmed by steady-state optical properties, which further evolves to triplet due to the high efficiency in this process (Φ_isc_ = 97%) caused by the heavy-atom effect.

**Figure 8:**
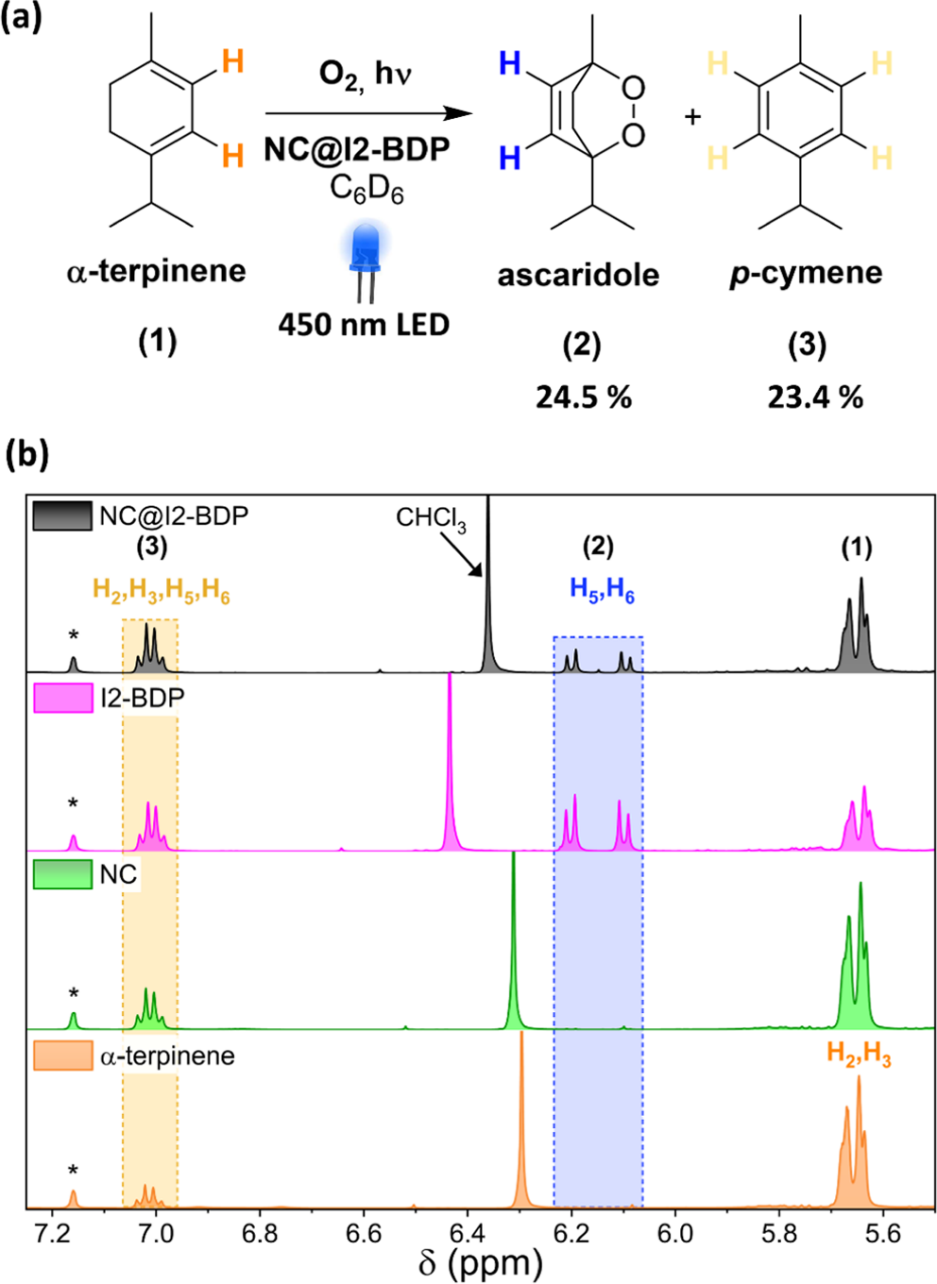
(a) Scheme of the catalyzed photooxidation reaction of α-terpinene by the NC@I2-BDP nanohybrid under visible light. (b) ^1^H NMR spectra of the crude reactions in C_6_D_6_ using different photocatalysts and the control; CHCl_3_ was used as the internal standard.

Thus, the efficient population of the I2-BDP triplet, as revealed by ^1^O_2_ phosphorescence measurements in NC@I2-BDP can be successfully used as an energy transfer photocatalyst for the oxidation of α-terpinene under visible-light excitation with a conversion of ca. 48%. α-Terpinene was purified by column chromatography before use (Figure S23). Interestingly, ascaridole (24.5%) was obtained together with the typical conversion of terpinene to *p*-cymene (23.4%), quantified by ^1^H NMR (Figure 8). Although the selectivity of the reaction was low, we demonstrated that NC@I2-BDP can be used as a photocatalyst, specifically, for the synthesis of ascaridole, using a small amount of NC (0.1 nmol), 2 h of irradiation under visible-light excitation, and an O_2_-saturated air atmosphere. Control experiments revealed that in the absence of NC and photocatalyst, ascaridole was not formed, and *p*-cymene was obtained with ca. 20%. Although I2-BDP produced higher conversion and ascaridole formation, the organic photosensitizer could not be recovered from the medium, and NC could be used as a heterogeneous photocatalyst. This could have implications in different areas, such as organic synthesis and pharmaceutical development, given the significance of ascaridole in certain medicinal contexts.

## Conclusions

Efficient deactivation of the CsPbBr_3_ NC excited state by the anchored BODIPY (BDP, I2-BDP) in NC@BODIPY nanohybrids was confirmed by using steady-state and time-resolved fluorescence combined with fs-TA spectroscopy. The presence of two iodine atoms directly appended to the BODIPY core in I2-BDP has a significant impact on dye photophysics after selective photoexcitation of NC. Our findings revealed the occurrence of mainly FRET (82%) and charge-mediated triplet energy transfer (64%) sensitization for NC@BDP and NC@I2-BDP, respectively. In the case of NC@I2-BDP, the charge-separated state was confirmed by (i) the detection of an ultrafast hot hole extraction component, (ii) the presence of a positive band at 544 nm assigned to I2-BDP^+•^, (iii) spectroelectrochemistry, and (iv) theoretical calculations of the radical cation species. The efficient generation of the I2-BDP triplet excited state, validated through singlet oxygen measurements, enabled the use of NC@I2-BDP as an energy transfer photocatalyst for the oxidation of α-terpinene to ascaridole with a moderate yield.

Herein, we have demonstrated that the functionalization of CsPbBr_3_ NC with photoactive BODIPYs, such as BDP and I2-BDP, can modulate the photophysics of the corresponding NC@BODIPY nanohybrid. The iodine substituents of I2-BDP played a key role in the photosensitization of the dye triplet. These findings pave the way for the use of efficient hybrid materials in light-energy conversion and photocatalytic applications.

## Supporting Information Available

Corresponding materials, methods, instrumentation, models, tables, and figures (PDF)
