# Supplementary material for: Engineering Metal Halide Perovskite Nanocrystals with BODIPY Dyes for Photosensitization and Photocatalytic Applications
Source: J Am Chem Soc. 2024 Apr 4;146(21):14479–92. doi: 10.1021/jacs.3c14335 (PMC11140745; doi:10.1021/jacs.3c14335)
Supplement: Supplementary file 1 — ja3c14335_si_001.pdf [file ja3c14335_si_001.pdf]

Supporting Information for:

Engineering metal halide perovskite nanocrystals with BODIPY dyes for photosensitization and photocatalytic applications

Alejandro Cortés-Villena,[a] Delia Bellezza,[a] Carla Cunha,[b] Ignacio Rosa-Pardo,[a] Álvaro Seijas-Da Silva,[a] João Pina,[b] Gonzalo Abellán,[a] J. Sérgio Seixas de Melo,[b]* Raquel E. Galian[a]* and Julia Pérez-Prieto.[a]*

[a] Institute of Molecular Science, University of Valencia, c/Catedrático José Beltrán Martínez 2, 46980 Paterna, Valencia, Spain.

[b] CQC-IMS, Department of Chemistry, University of Coimbra, Coimbra P-3004-535, Portugal.

Address correspondence to: sseixas@ci.uc.pt, raquel.galian@uv.es and julia.perez@uv.es

Table of Contents

**Materials**	**S4**
Perovskite nanocrystals	**S4**
BODIPY dyes	**S4**
Nanohybrids	**S4**
**Methods**	**S5-S6**
Synthesis of NCs	**S5**
Synthesis of BODIPY dyes	**S5-S6**
Preparation of NC@BODIPY nanohybrids	**S6**
**Instrumentation**	**S7-S8**
**Determination of energy levels**	**S9**
**Fitting model for TCSPC data**	**S10**
**Rate constant estimation**	**S11**
**Fitting model for fs-TA data**	**S12**
**Global analysis for fs-TA data**	**S13**
**Energy transfer mechanism**	**S14**
**Quantum electronic calculations**	**S15-S16**
**Table S1.** Potentials and energetics.	**S9**
**Table S2.** Time constants from TCSPC data. Selective excitation to NC.	**S10**
**Table S3.** Time constants from TCSPC data. Selective excitation to BODIPY dyes.	**S10**
**Table S4.** Time constants from fs-TA data. Selective excitation to NC.	**S12**
**Table S5.** Time constants from fs-TA data. Selective excitation to BODIPY dyes.	**S12**
**Table S6.** Time constants from fs-TA data obtained by GA. Selective excitation to NC.	**S13**
**Table S7.** Calculated ground-state absorption of BODIPY dyes.	**S15**
**Table S8.** Calculated transient-absorption of BODIPY dyes.	**S15-S16**
**Figure S1.** TEM image of NCs.	**S17**
**Figure S2.** NMR properties of BDP dye.	**S18**
**Figure S3.** NMR properties of I2-BDP dye.	**S19**
**Figure S4.** Electrochemical properties of NC, BDP and I2-BDP dyes.	**S20**
**Figure S5.** Steady-state normalized absorption and emission spectra of NC, BDP and I2-BDP dyes.	**S21**
**Figure S6.** Comparative of PL decay traces of NC and NC@BDP under 420 nm excitation.	**S22**
**Figure S7.** Comparative of PL decay traces of NC and NC@I2-BDP under 450 nm excitation.	**S23**
**Figure S8.** Comparative of PL decay traces of BODIPY dyes and nanohybrids.	**S24**
**Figure S9.** fs-TA properties of BODIPY dyes.	**S25**
**Figure S10.** FTIR of nanohybrids along with controls.	**S26**
**Figure S11.** DAS for NC and NC@BDP nanohybrid.	**S27**
**Figure S12.** fs-TA spectra of NC and NC@I2-BDP nanohybrid and kinetic trace of NC@I2-BDP probed at 560 nm.	**S28**
**Figure S13.** ns-TA properties of BODIPY dyes.	**S29**
**Figure S14.** Spectroelectrochemistry of I2-BDP dyes.	**S30**
**Figure S15.** Optimized structures of BODIPY dyes.	**S31**
**Figure S16.** Calculated absorption spectra and natural transition orbitals of BODIPY dyes.	**S31**
**Figure S17.** Natural transition orbitals of BODIPY dyes for emission.	**S32**

**Figure S18.** Optimized structures for neutral, radical anion and cation of I2-BDP dyes.	**S32**
**Figure S19.** Calculated absorption spectra and natural transition orbitals of I2-BDP dyes dyes for radical cation and anion structures.	**S33**
**Figure S20.** Steady-state normalized PLE spectra of nanohybrids along with controls.	**S34**
**Figure S21.** Singlet oxygen measurements of nanohybrids along with controls.	**S35**
**Figure S22.** Phosphorescence spectra of BODIPY dyes.	**S36**
**Figure S23.** NMR properties of purified α-terpinene.	**S36**
**References**	**S37**

Materials and methods

Perovskite nanocrystals

Cesium carbonate (Cs_2CO_3, 99%, Sigma Aldrich), oleic acid (OA, 90%, Alfa Aesar), 1-octadecene (1-ODE, 90%, Alfa Aesar), lead bromide ($PbBr_2$, 99.999%, Sigma Aldrich), oleylamine (OAm, 70%), toluene (Tol, Essent-Q, Scharlab) and methyl acetate anhydrous (MeOAc, 99.5%, Sigma Aldrich).

BODIPY dyes

All chemicals and solvents obtained from commercial sources were used without further purification unless specified. 2,4-dimethylpyrrole (>97%, TCI), 4-formylbenzoic acid (97%, Sigma Aldrich), trifluoroacetic acid (TFA, ≥99%, Sigma Aldrich), 2,3-dichloro-5,6-dicyano-p-benzoquinone (DDQ, 98%, Sigma Aldrich), boron trifluoride diethyl etherate ($BF_3·OEt_2$, 99.7+%, Alfa Aesar), iodine (>99.8%, Sigma Aldrich), iodic acid (99.99%, Sigma Aldrich), ethanol absolute (EtOH, Essent-Q, Scharlab), sodium thiosulfate ($Na_2S_2O_3$, 99%, Sigma Aldrich), dichloromethane anhydrous (DCM, 98+%, Alfa Aesar), methanol (MeOH, HPLC grade, Scharlab) and sodium sulfate anhydrous (Na_2SO_4, >99%, Fisher). Triethylamine (TEA, 99%, Sigma Aldrich) was dried by refluxing with CaH_2 and distillated. Deuterated DMSO (DMSO-d_6) with at least 99% isotope content (Eurisotop) was used for NMR measurements. Columns chromatography was conducted in self-packed glass columns of different sizes with silica gel (particle size: 40 – 60 µm, Merck).

Nanohybrids

Hexane anhydrous (Hx, 95%, Sigma Aldrich).

Synthesis of NCs

The $CsPbBr_3$ perovskite nanocrystals (NCs) were synthesized accordingly by a previously reported hot injection method with slight modifications.[1] All experiments were carried out using a Schlenk line. First of all, a solution of cesium oleate (0.125 M in 1-ODE) was prepared by mixing cesium carbonate (407 mg, 1.24 mmol) and oleic acid (1.25 mL, 3.53 mmol) in 1-octadecene (20 mL) in a 50 mL three-neck round-bottom flask and the mixture was degassed under vacuum (0.49 mbar) at 120 ˚C for 1 h. Then, the mixture was heated up to 150 ˚C under N_2 atmosphere until Cs_2CO_3 was completely dissolved (ca. 30 min) obtaining a clear solution. Afterwards, the solution was slowly cooled down to room temperature and stored under N_2 atmosphere for being used. On the other hand, for the synthesis of NCs, lead bromide (72 mg, 0.196 mmol), oleylamine (0.71 mL, 1.5 mmol) and oleic acid (0.53 mL, 1.5 mmol) were loaded into a 50 mL three-neck round-bottom flask with 1-octadecene (4.8 mL) and degassed under vacuum (1 mbar) at 100 ˚C for 30 min. Then, the valve of the Schlenk line was changed to N_2 flow and after 15 min, it yielded a colorless solution. At the same time, the cesium oleate solution was heated up to 120 ˚C under N_2 atmosphere. After complete solubilization of the lead bromide salt, the reaction temperature was raised to 190 ˚C and cesium oleate solution (0.6 mL, 0.075 mmol) was swiftly injected through a purged syringe and, 5 s later, the reaction was quenched through an ice/water bath (bath temperature ca. -2 ˚C) and the NCs were finally subjected to purification. The crude dispersion was first diluted with 5 mL of toluene and transferred to a 50 mL centrifuge tube to initiate the isolation process. The crude was first centrifuged at 2500 rpm for 10 min (15 ˚C). The supernatant was carefully pipetted out and transferred to another 50 mL centrifuge tube. Methyl acetate anhydrous (8 mL) was added to the supernatant to help the precipitation of smaller NCs and was secondly centrifuged at 5000 rpm for 10 min (15 ˚C). This second precipitate was finally redispersed in 2 mL of toluene. This dispersion was finally filtrated out through a 0.2 μm membrane filter to yield highly-colloidal, homogeneous $CsPbBr_3$ NCs dispersion (see Scheme 1). Based on the molar extinction coefficient for 6.5 nm-sized $CsPbBr_3$ NCs, a concentration of ca. 1.2 μM was obtained.

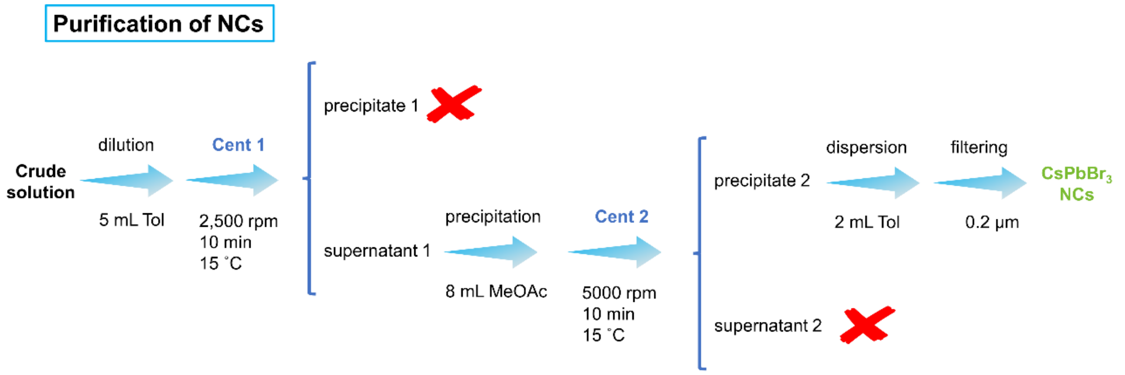

Scheme 1. Purification strategy of the as-synthesized $CsPbBr_3$ NCs.

Synthesis of BODIPY dyes

8-(4-carboxyphenyl)-1,3,5,7-tetramethyl-4,4-difluoro-4-bora-3a,4a-diaza-s-indacene (BDP): BDP dye was synthesized according to reported procedures.[2] First, 4-carboxybenzaldehyde (0.6 g, 4.0 mmol) was added to a 500 mL round-bottom flask in 200 mL of dichloromethane anhydrous. Then 2,4-dimethyl pyrrole (8.97 mmol, 0.92 mL) was added followed by the addition of TFA (30 μL) and the solution was stirred overnight under Ar at room temperature. After addition of a solution of DDQ (4 mmol, 0.9 g) in DCM (100 mL) to the reaction mixture, stirring was continued for 10 minutes. Then 8 mL of TEA and 8 mL of $BF_3{\cdot}OEt_2$ were successively added. After 12 hours, the reaction mixture was treated with water (300 mL). Then the mixture was extracted into the DCM. The organic layer was dried over anhydrous Na_2SO_4. The solvent was evaporated and the residue was purified by silica gel columns chromatography (DCM : MeOH = 30:1, v/v and Hex : AcOEt = 3:1 v/v). The BDP was obtained as a red solid with a Chemical yield of 13% (Scheme 2).

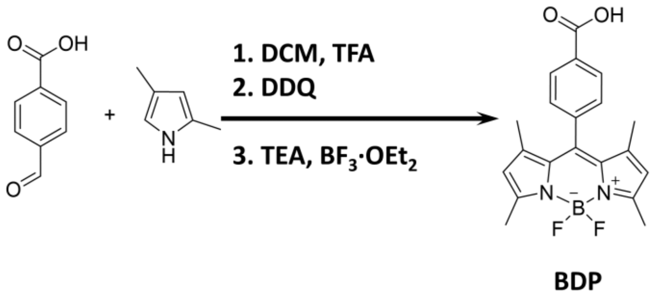

Scheme 2. Synthesis of the BDP dye.

8-(4-carboxyphenyl)-2,6-diiodo-1,3,5,7-tetramethyl-4,4-difluoro-4-bora-3a,4a-diaza-s-indacene (I2-BDP): I2-BDP was also synthesized according to a reported method.[3] To the solution of BDP (100 mg, 0,27 mmol) in 20 mL of EtOH, iodine (171 mg, 0,68 mmol) and iodic acid (95 mg, 0,54 mmol) were added dissolved in 1 mL of water. The reaction mixture was stirred at 60 ˚C for 2 hours. Then, a saturated $Na_2S_2O_3$ solution was added, and EtOH was removed under reduced pressure. The residue was extracted with dichloromethane. The organic layer was dried over anhydrous Na_2SO_4. The solvent was evaporated and the residue was purified by silica gel column chromatography using DCM : MeOH = 30:1, v/v) as eluent to give rise to I2-BDP as deep red solid. Chemical yield: 80% (Scheme 3).

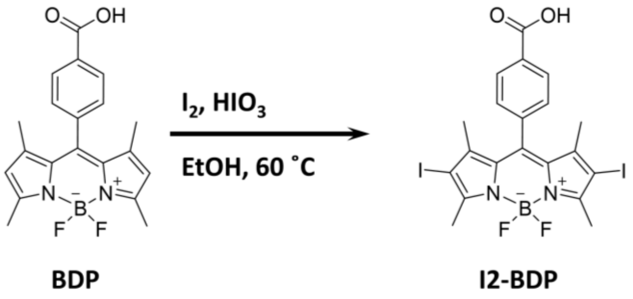

Scheme 3. Synthesis of the I2-BDP dye.

Preparation of NCs@BODIPY nanohybrids

An aliquot of the as-synthetized NCs in toluene was evaporated and then redispersed in hexane because of the greater solubility of both dyes in toluene compared to hexane. Overall, dye powders (484 nmol) were added to a 1.5 mL NCs dispersion in hexane (0.24 nmol) optically-matched at 0.64 OD at 450 nm in 1 cm pathlength cuvette in a molar ratio of ca. 2000. The mixtures were stirred for 15 min and then filtrated through a 0.2 µm membrane filter to remove the unreacted solid and/or formed aggregates. The dyes in the final hexane dispersion were bound/encapsulated to NCs surface.

Instrumentation

Steady-state UV-vis absorption spectroscopy. Steady-state UV-vis absorption spectra were recorded on a UV/Vis/NIR Perkin Elmer Lambda 1050 spectrophotometer equipped with deuterium and tungsten halogen light sources and Peltier-controlled InGaAs and PbS detectors covering from 175 to 3300 nm. All spectra were recorded using a 1 cm path length quartz cuvette at room temperature.

Steady-state photoluminescence spectroscopy. Stationary photoluminescence spectra (PL: emission and PLE: excitation) were recorded on a FLS1000 photoluminescence spectrometer (Edinburgh Instruments) equipped with a 450 W ozone free continuous xenon arc lamp and a photomultiplier (PMT-980) detector in cooled housing with extended spectral range from 185 to 980 nm. For singlet oxygen ($^1O_2$) phosphorescence measurements, a NIR-PMT detector (Hamamatsu Photonics) with a spectral response ranging from 950 to 1700 nm was employed. All spectra were recorded using a 1 cm path length quartz cuvette at room temperature.

Photoluminescence quantum yield. Absolute photoluminescence quantum yields (Φ_{PL}) were recorded on a FLS1000 photoluminescence spectrometer equipped with an integrating sphere system with a reflectance higher than 99% in the range 400-1500 nm. Sample solutions with 0.1 OD excitation wavelength was used to minimize re-absorption effects. For reference, the neat solvent with exactly the same volume was used. All spectra were recorded using a 1 cm path length quartz cuvette at room temperature.

Time-resolved photoluminescence spectroscopy. Time-resolved photoluminescence measurements were recorded on a FLS1000 photoluminescence spectrometer through the time-correlated single photon counting (TCSPC) technique coupled with a picosecond pulsed supercontinuum white light laser (SuperK, repetition rate: 9.8 MHz, NKT Photonics) and a microchannel plate (MCP-900) detector in cooled housing with spectral range from 200 to 850 nm in the picosecond domain. A Ludox solution (0.1 OD at excitation wavelength) was used as instrument response function (IRF). The IRF is about 150 ps for all excitation used in our setup. All spectra were recorded using a 1 cm path length quartz cuvette at room temperature.

Nanosecond transient absorption spectroscopy. Nanosecond transient absorption spectroscopy (ns-TAS) employing spectral and time-resolved transient species absorption measurements were performed on a laser flash photolysis spectrometer (LP980-KS, Edinburgh Instruments) equipped with a Quanta-Ray INDI Nd:YAG laser (Spectra Physics) with a parametric optical oscillator (primoScan BB, Spectra Physics). The laser output power was 5 mJ for the pumping wavelengths and the instrumental response function was 10 ns. Spectral measurements were made using an ICCD camera (Andor DH320T) that integrates for 500 ns (gate width) with respect to the indicated delay of the laser pulse and with a 150W Xe pulsed lamp. The kinetics were obtained with a photomultiplier detector and the pulsed Xe lamp. Optically-matched solutions were used in a 1 cm septum-stoppered quartz cuvettes (standard cross-beam geometry).

Femtosecond transient absorption spectroscopy. Femtosecond transient absorption spectroscopy (fs-TAS) experiments were performed on a Helios spectrometer (Ultrafast Systems) with an instrumental response function of ~250 fs. The instrumental response function of the system was assumed to be equal to that of the pump–probe cross correlation determined from the measurement of the instantaneous stimulated Raman signal from the pure solvent (in a 2 mm cuvette). To avoid photodegradation, the solutions were stirred during the experiments or in movement using a motorized translating sample holder. An optical delay line of up to 7 ns was employed. An excitation flux of 0.05 $mJcm^{-2}$ was employed for all measurements. Data analysis was made on Surface Xplorer and GloTarAn software.[4]

Transmission electron microscopy. Transmission electron microscopy (TEM) was performed on a HITACHI HT7800 microscope with a filament of LaB_6 operated at 100 keV.

Nuclear magnetic resonance. The ^1H (500 MHz) and ^{13}C (126 MHz) spectra were recorded at 298 K on a Bruker instrument. Chemical shifts (δ) are reported in ppm relative to TMS (δ = 1H = 0.0 ppm) and coupling constants (*J*) are given in Hertz (Hz). The chemical shift range for each spectrum was calibrated using the residual solvent signals as internal reference.

Attenuated total reflectance-Fourier transform infrared spectroscopy. The ATR-FTIR spectra were collected on a Bruker alpha II FTIR spectrometer in the 4000–400 cm^{-1} range. A few drops were added, and the solvent was completely dried.

Electrochemical measurements. Redox properties characterization was performed on an Autolab 128N potentiostat/galvanostat using a three-electrode system. Cyclic voltammetry (CV) experiments were carried out in 0.13 M tetrabutylammonium tetrafluorophosphate ($TBAPF_6$) solution in a mixture of dried

ACN:toluene (1:3 v/v) for both dyes and NCs, by using a glassy carbon working electrode, Pt wire counter electrode and Ag as pseudo-reference electrode. Concentration of both dyes and NCs were 1.13 mM and ca. 0.1 μM, respectively. The measurements were performed at room temperature partially deaerating the electrolyte solution by bubbling N_2 for 1 min. Experiments were performed under air conditions, with a scan rate of 200 mV/s. To determine the electrochemical bandgaps, potentials were referred to the Fc/Fc^+ redox couple using 0.4 mM solutions of ferrocene in 0.13 M $TBAPF_6$ in the mixture of solvents.

Spectroelectrochemical measurements. A three-electrode electrochemical system comprised of an Ag wire, Pt wire and ITO substrate as the pseudo-reference, counter and working electrode was used in a quartz home-made cell. The I2-BDP dye was drop-casted onto the transparent ITO substrate. An electrolyte solution of 0.5 M $TBAPF_6$ was prepared in ACN. This quartz cell was placed along the optical path of the UV-vis spectrophotometer described above. A chronoamperometry measurement of 2 min was applied for a given potential during the acquisition of an UV-vis spectrum.

Determination of energy levels

Estimation of the band edge positions (VB and CB) on NC with respect to frontier molecular orbitals (HOMO and LUMO) of both BDP and I2-BDP was carried out by using the following equations:

$$E_{HOMO/VB} = -[V_{ox} - V(Fc/Fc^{+}) + 4.8]\ eV \qquad (S1)$$

$$E_{LUMO/CB} = -[V_{red} - V(Fc/Fc^{+}) + 4.8]\ eV \qquad (S2)$$

V_{ox} and V_{red} are the irreversible oxidation and reduction peak values determined in CV measurements, $V(Fc/Fc^{+})$ is the oxidation potential at semi-wave of Fc/Fc^{+} couple external reference and 4.8 is the energy term of Fc/Fc^{+} against vacuum.

Table S1. Potentials and energetics determined from CV measurements.

Features / Sample	V_{ox} (V)	E_{HOMO}/VB (eV)	V_{red} (V)	E_{LUMO}/CB (eV)	ΔE^{CV} (eV)[a]	ΔE^{Opt} (eV)	E_{LUMO}/CB (eV)[b]
BDP	+ 0.86	-5.66	-1.63	-3.17	2.49	2.43	-3.23
I2-BDP	0.99	-5.79	-1.58	-3.22	2.57	2.26	-3.53
NC	+1.00	-5.80	-1.53	-3.27	2.53	2.45	-3.35

[a] Obtained through $E_{LUMO/CB}$ - $E_{HOMO/VB}$.

[b] Estimated values considering subtraction between $E_{HOMO/VB}$ and ΔE^{Opt}.

Fitting model for TCSPC data

We used multiexponential function fittings for decay traces obtained by TCSPC. The following fitting equation is:

$$S(t) = A_1 e^{(-t/\tau_1)} + A_2 e^{(-t/\tau_2)} + A_3 e^{(-t/\tau_3)} \otimes IRF \qquad (S4)$$

where S(t) is the PL signal at time t, τ_1, τ_2 and τ_3 are the time constants for surface non-radiative, direct electron-hole radiative recombination and trap-assisted recombination, respectively, and A_1, A_2 and A_3 are their corresponding amplitudes. IRF is the convoluted instrumental response function which accounts for the excitation pulse width, detector and electronic response time, and pulse temporal dispersion.

The relative amplitude or lifetime-weighted contribution (A_i %) is calculated based on the following formula:

$$A_1 = \frac{(A_1\tau_1)}{\sum_i (A_i\tau_i)} x100 \qquad (S5)$$

The average PL decay lifetime (τ_{av}) is calculated accordingly the following equation:

$$\tau_{av} = \frac{\sum_i (A_i\tau_i^2)}{(A_i\tau_i)} \qquad (S6)$$

The fitting parameters are listed in Table S2 and S3.

Table S2. Summarized time constant parameters obtained from TCSPC measurements. Selective excitation to NCs.

Features / Sample	λ_{exc} (nm)	λ_{em} (nm)	τ_1 ± sd (ns)	A_1 (%)	τ_2 ± sd (ns)	A_2 (%)	τ_3 ± sd (ns)	A_3 (%)	τ_{av} ± sd (ns)
NC	420	502	0.81 ± 0.01	13	3.42 ± 0.02	67	11.71 ± 0.12	20	4.66 ± 0.05
NC@BDP			0.15 ± 0.01	9	2.6 ± 0.02	56	7.0 ± 0.07	35	3.97 ± 0.04
		570	0.11 ± 0.01	2	3.16 ± 0.01	77	8.97 ± 0.07	21	4.40[b] ± 0.15
NC	450	502	0.7 ± 0.01	21	2.9 ± 0.03	60	11.1 ± 0.17	19	4.01 ± 0.07
NC@I2-BDP[a]			-	-	-	-	-	-	-

[a] Decay trace limited by setup resolution.
[b] Calculated taking into account only decay components.

Table S3. Summarized time constant parameters obtained from TCSPC measurements. Selective excitation to BODIPY dyes.

Features / Sample	λ_{exc} (nm)	λ_{em} (nm)	τ_1 ± sd (ns)	A_1 (%)	τ_2 ± sd (ns)	A_2 (%)	τ_{av} ± sd (ns)
BDP	515	570	2.27 ± 0.01	100			2.27 ± 0.01
NC@BDP			2.28 ± 0.01	91	5.03 ± 0.16	9	2.52 ± 0.03
I2-BDP	550	600	0.22 ± 0.01	86	3.32 ± 0.06	14	0.25 ± 0.02
NC@I2-BDP			0.23 ± 0.01	90	2.86 ± 0.07	10	0.50 ± 0.01

Rate constant estimation

An approximate way to calculate rate constants is by using the following equation:

$$k = {}^{1}\!/_{\tau_{NCs@BODIPY}} - {}^{1}\!/_{\tau_{NC}} \qquad \text{(S3)}$$

where k is the rate constant of the corresponding process, $\tau_{NC@BODIPY}$ is the time constant of NC in presence of BODIPY dyes and τ_{NC} is the time constant of pristine NC measured at the same conditions.

Fitting model for fs-TA data

Multiexponential decay functions were used to describe the exciton/charge carrier dynamics monitored at XB for pristine NC and NC@BODIPY nanohybrids and BODIPY dyes in the following form:

$$\Delta OD = \sum_{i=1}^{n} A_i e^{\left(-t/\tau_i\right)} \qquad \text{(S7)}$$

where ΔOD is the optical density difference, A_i and τ_i are the amplitude and time constant of the *ith* component, respectively. The fitting parameters are listed in Table S4.

Table S4. Summarized time constants obtained from fs-TA measurements. Selective excitation to NC. The single-wavelength analysis was performed in Surface Xplorer software. An infinite offset was needed in the analysis to account for non-decay data. Standard deviation of residuals shown.

Features / Sample	λ_{pump} (nm)	λ_{probe} (nm)	τ_1 ± sd (ps)[a]	τ_2 ± sd (ps)	A_2 (%)	τ_3 ± sd (ns)	A_3 (%)	τ_4 ± sd (ns)	A_4 (%)	σ
NC	420	498	0.28 ± 0.04	151 ± 52	5	3.57 ± 0.09	95			3.78 10^{-5}
NC@BDP			0.29 ± 0.07	26.38 ± 0.99	85	7[b]	15			6.2 10^{-5}
NC	450		0.22 ± 0.08	206 ± 61	5	3.57 ± 0.12	95			3.31 10^{-5}
NC@I2-BDP			0.18 ± 0.08	9.46 ± 0.39	55	0.27 ± 0.08	11	2.84 ± 0.23	34	3.27 10^{-5}

[a] Time constant related to rise time of XB.
[b] Fixed value taken from TCSPC data.

Table S5. Summarized time constants obtained from fs-TA measurements for BODIPY dyes. The single-wavelength analysis was performed in Surface Xplorer software. An infinite offset was needed in the analysis to account for non-decay data. Standard deviation of residuals shown.

Features / Sample	λ_{pump} (nm)	λ_{probe} (nm)	τ_1 ± sd (ps)[a]	A_2 (%)	τ_2 ± sd (ns)	A_3 (%)	σ
BDP	515	525	42.98 ± 4.10	23	2.63 ± 0.10	77	1.93 10^{-5}
I2-BDP	550	570	198 ± 1	100			2.34 10^{-5}

Global analysis for fs-TA data

We applied Global Analysis (GA) to fs-TA data for extracting information from multiple datasets simultaneously. This is very convenient when the improvement in signal to noise ratio and data quality is pursued and wanted more reliable results. The entire analysis was carried out with GloTarAn software.[4]

First of all, a singular value decomposition (SVD) procedure was employed as a pre-analysis tool to estimate the number of linearly-independent components in the mxn matrix:

$$\Psi_{mxn} = U_{mxm} S_{mxn} W_{nxn}^{T} \qquad \text{(S8)}$$

being U and W orthogonal matrices, containing the left and right singular vectors, respectively, and S is a matrix which contain the singular values or eigenvalues. The left and right singular vectors represent the time- and spectral-dependence of the particular components and are particularly helpful in determining the number of components clearly distinct from the noise and quality of the results.

The basic form of the kinetic model describing the measured data Ψ as a function of time and spectral $\Psi(t,\lambda)$ can be described as follows:

$$\Psi(t,\lambda) = \sum_{i=1}^{n_{comp}} e^{-k_i t} \epsilon_i(\lambda) \otimes IRF \qquad \text{(S9)}$$

where k_i is the rate of exponential decay of component i, $\epsilon_i(\lambda)$ is the amplitude of the concentration profiles and $\otimes$ IRF is the convolution with the instrumental response function.

To fit the data, we based on a parallel model because of the complexity in the photophysical picture, where every component decays independently resulting in decay associated spectra (DAS). The basic principle of DAS in GA is to decompose simultaneously by using the kinetic model the time-resolved spectra into a set of kinetic components, each associated with a specific decay time constant.

Table S6. Summarized time constants obtained from GA. Selective excitation to NCs. The GA was performed in GloTarAn software. The last component was always fixed during analysis.

Features / Sample	λ_{pump} (nm)	τ_1 ± sd (ps)	τ_2 ± sd (ps)	τ_3 ± sd (ns)	τ_4 ± sd (ns)	τ_5 ± sd (μs)
NC	420	0.29 ± 0.01	191 ± 18	3.23 ± 0.06	11[a]	
NC@BDP		0.28 ± 0.01	25.82 ± 0.13	8.22 ± 0.51	5000[b]	
NC	450	0.21 ± 0.01	293 ± 23	3.37 ± 0.06	11[a]	
NC@I2-BDP		0.16 ± 0.01	10.36 ± 0.15	0.20 ± 0.01	2.42 ± 0.05	49[c]

[a] Fixed value taken from TCSPC data.
[b] Fixed value considering the triplet nature due to small singlet oxygen yield.
[c] Fixed value taken from ns-TA.

Energy transfer mechanism

Non-radiative energy transfer (ET) can occur through two widely-known different mechanisms: Förster resonance ET (FRET) or dipole-dipole coupling and Dexter ET (DET) or electronic exchange.[5] Here, we will only consider FRET mechanism (see Main text). The formalism for the FRET mechanism, expressed in terms of the rate constant of ET (k_{ET}) can be described as follow:

$$k_{FRET} = \frac{1}{\tau_D}(\frac{R_0}{r_{DA}})^6 \qquad \text{(S10)}$$

being $\tau_D$the donor lifetime in absence of energy transfer, R_0 is the Förster distance and r_{DA} is the distance between the donor and acceptor. Because it is a dipole-dipole coupling mechanism, the R_0 is defined as:

$$R_0^6 = \frac{\Phi_D \kappa^2 9000(\ln{(10)})}{128\pi^5 N n^4} J(\varepsilon_A) \qquad \text{(S11)}$$

which accounts for the donor quantum yield in absence of energy transfer (Φ_D), dipole orientation (κ, with a value of 2/3 usually used for NCs), solvent refractive index (n, 1.497 for toluene) and spectral overlap between donor emission and acceptor absorption ($J(\varepsilon_A)$). This $J(\varepsilon_A)$ is then defined as:

$$J(\lambda) = \int_0^\infty F_D(\lambda)\varepsilon_A(\lambda)\lambda^4 d\lambda \qquad \text{(S12)}$$

where $F_D(\lambda)$ is the fluorescence integral of the donor and $\varepsilon_A(\lambda)$ is the molar extinction coefficient of the acceptor.

The efficiency of FRET (Φ_{FRET}) between the donor emission and acceptor absorption in a single donor-acceptor pair at a fixed distance can be calculated as:

$$\Phi_{FRET} = \frac{{R_0}^6}{{R_0}^6 + r^6} \qquad \text{(S13)}$$

Quantum electronic calculations

All theoretical calculations were of the DFT type, carried out using GAMESS-US version R3.[6] All the structures in the ground and excited state were fully optimized without any symmetry restriction using LC-BPBE (w=0.20 au^{-1}) level of theory in solvent toluene. The nature of the stationary points determined by geometry optimization were verified by vibrational frequency calculations carried out at the same level of theory. All the reported geometries correspond to minima. In TDDFT calculations of FC (Franck-Condon) excitations the dielectric constant of the solvent was split into a "bulk" component and a fast component, which is essentially the square of the refractive index. In "adiabatic" conditions only the static dielectric constant is used. A 6-31G** basis set was used in either DFT or TDDFT calculations.

The results obtained with the LC-BPBE(20) functional are essentially unscaled raw data from calculations; for the $S_0 \rightarrow S_n$ transitions, a small correction, which result in the subtraction of 0.05 eV, to account for the difference between zero point and the first vibronic level, was considered. For the resulting optimized geometries time dependent DFT calculations (using the same functional and basis set as those in the previously calculations) were performed to predict the vertical electronic excitation energies. Molecular orbital contours were plotted using ChemCraft 1.7 program. Frequency analysis for each compound were also computed and did not yield any imaginary frequencies, indicating that the structure of each molecule corresponds to at least a local minimum on the potential energy surface.

Table S7. Selected electronic excitation energies (eV) and oscillator strengths (*f*), configurations of the low-lying excited states of the BDP and I2-BDP calculated by LC-BPBE(w=0.2), based on the optimized ground state geometries in toluene.

**Compound**	**eV, (nm)[a]**		***f*[b]**	**Transition[c]**
**BDP**	$S_0 \rightarrow S_1$	2.731 (454)	0.671	HOMO → LUMO
	$S_0 \rightarrow S_2$	3.471 (357)	0.060	HOMO-1 → LUMO
	$S_0 \rightarrow S_3$	3.756 (330)	0.069	HOMO-2 → LUMO
**I2-BDP**	$S_0 \rightarrow S_1$	2.646 (469)	0.758	HOMO → LUMO
	$S_0 \rightarrow S_2$	3.334 (372)	0.152	HOMO-2 → LUMO
	$S_0 \rightarrow S_3$	3.486 (356)	0.103	HOMO-1 → LUMO

[a] only the selected low-lying excited states are present; [b] oscillator strength; [c] only the main configurations are presented.

Table S8. Predicted electronic transitions ($S_1 \rightarrow S_n$) for I2-BDP (neutral, radical anion, and radical cation forms) obtained from TDDFT in toluene. The oscillator force (*f*) is reported together with the data obtained at the level theory of the DFT//LC-BPBE(w=0.2).

	$\mathbf{l_{nm}}$	***f*[b]**	$\mathbf{S_1 \rightarrow S_n}$
**Neutral**	633	0.006	$S_1 \rightarrow S_8$
	616	0.002	$S_1 \rightarrow S_9$
	585	0.375	$S_1 \rightarrow S_{10}$
	572	0.001	$S_1 \rightarrow S_{11}$
	534	0.000	$S_1 \rightarrow S_{12}$
	508	0.001	$S_1 \rightarrow S_{13}$

	484	0.002	$S_1 \rightarrow S_{14}$
	479	0.022	$S_1 \rightarrow S_{15}$
**Radical anion**	613	0.003	$S_1 \rightarrow S_6$
	609	0.002	$S_1 \rightarrow S_7$
	574	0.000	$S_1 \rightarrow S_8$
	522	0.000	$S_1 \rightarrow S_9$
	481	0.000	$S_1 \rightarrow S_{10}$
**Radical cation**	695	0.592	$S_1 \rightarrow S_{10}$
	576	0.144	$S_1 \rightarrow S_{11}$
	504	0.010	$S_1 \rightarrow S_{12}$
	488	0.022	$S_1 \rightarrow S_{13}$
	471	0.013	$S_1 \rightarrow S_{14}$

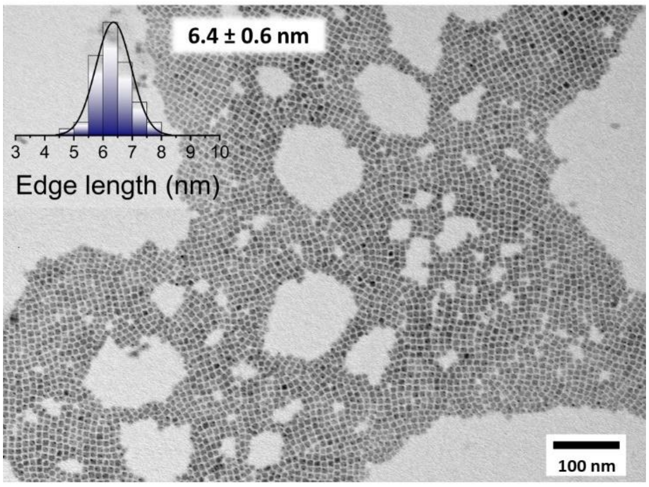

Figure S1. Representative TEM image of pristine NCs and size distribution histogram used in this study. 100 nanocrystals were accounted for the construction of histogram. The scale bar is 100 nm.

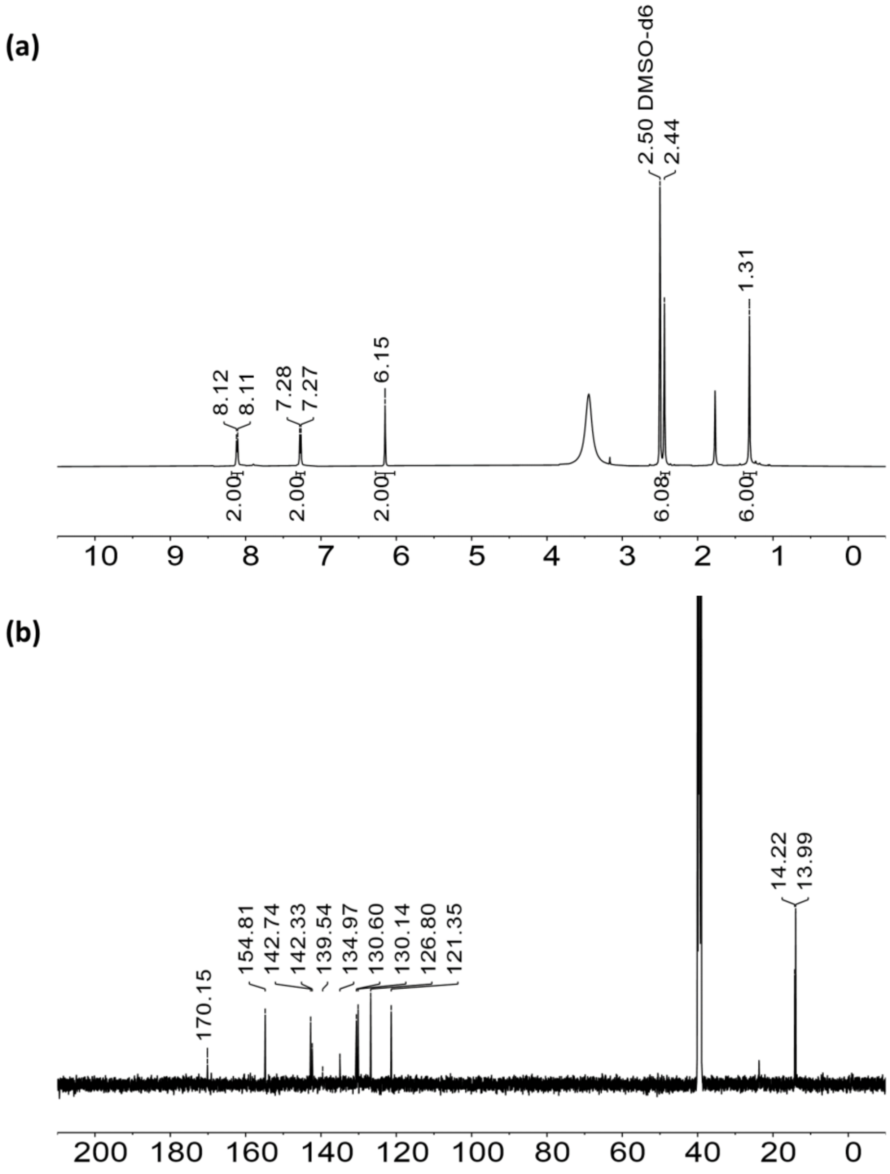

Figure S2. (a) ^{1}H-NMR and (b) ^{13}C-NMR spectra of BDP in DMSO-d_6.

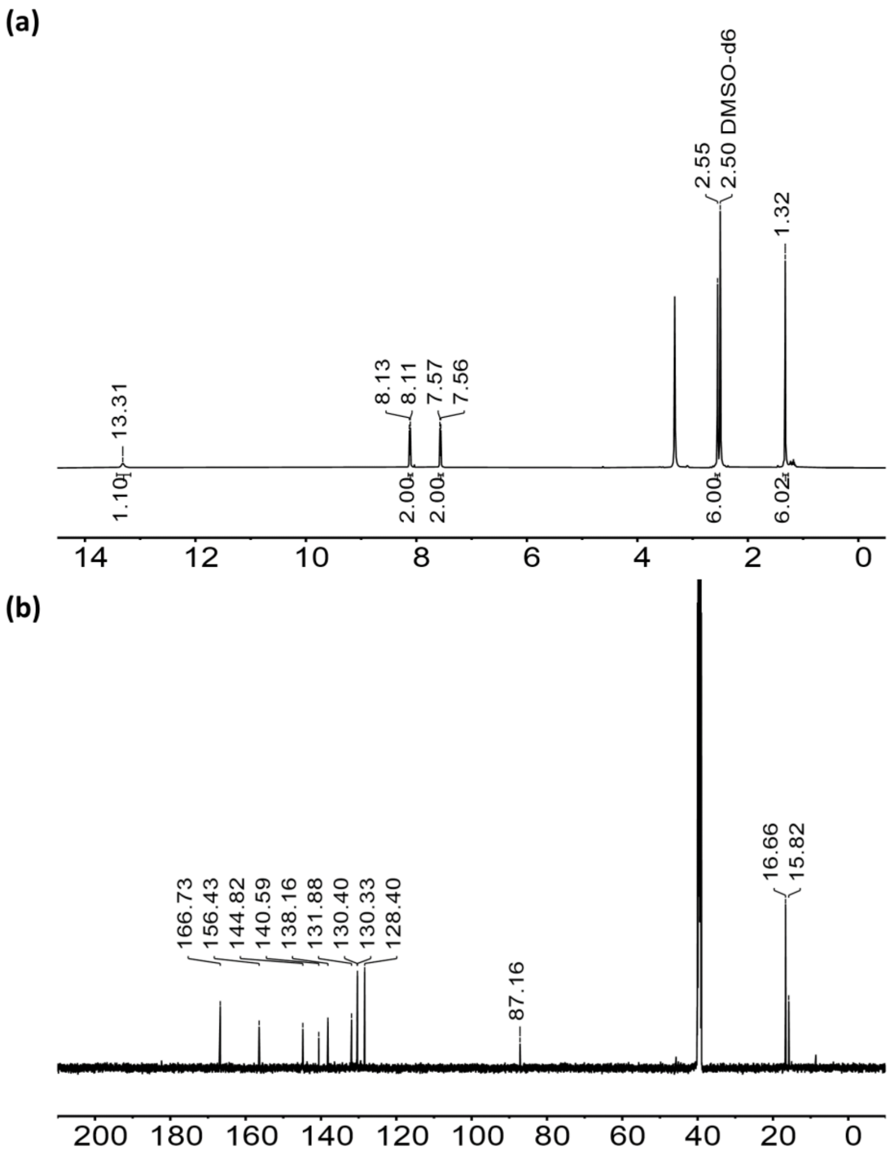

Figure S3. (a) ^{1}H-NMR and (b) ^{13}C-NMR spectra of I2-BDP in DMSO-d_6.

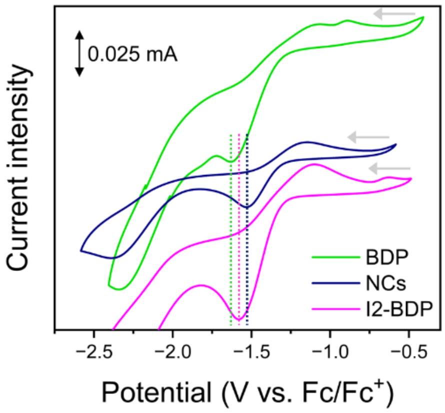

Figure S4. Cyclic voltammetry (CV) curves vs Fc/Fc+ redox couple. Cyclic voltammograms for cathodic region of NC and BODIPY dyes in a mixture of ACN/toluene (1:3 v/v). The dashed lines represent irreversible redox peaks. The scan rate is 0.2 Vs^{-1}. Arrows indicate the scan direction.

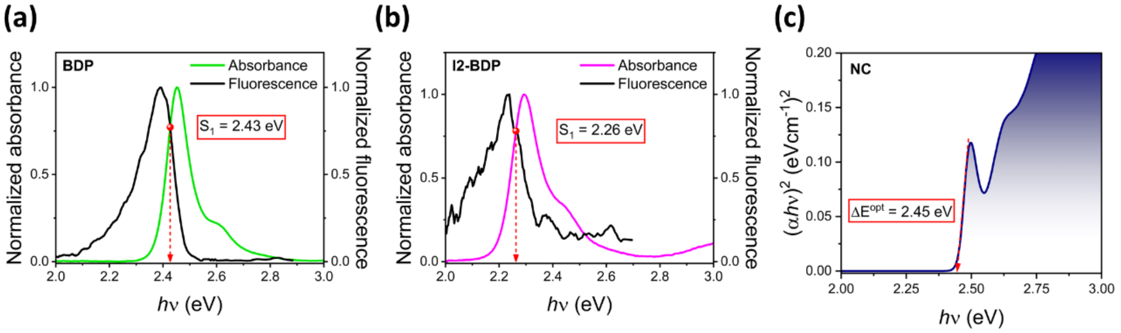

Figure S5. Steady-state normalized UV-vis absorption and fluorescence spectra of (a) BDP and (b) I2-BDP in toluene, respectively. The red point indicates the crossing point between both curves which determines the singlet state energy gap. (c) Tauc plot of pristine NC. The red dashed line is extrapolated to the x-axis for determination of the optical band gap. Excitation wavelength is 420 nm for (a), and 450 nm for (b).

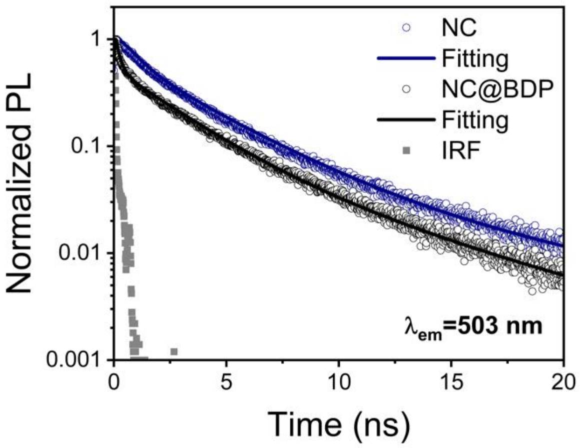

Figure S6. PL decay traces of pristine NC (blue curve) and NC@BDP (black curve) in toluene monitored at 502 nm for NC and NC@BDP upon 420 nm pulsed laser excitation (9.8 MHz). The instrument response function (IRF) was ca. 150 ps calculated at the FWHM.

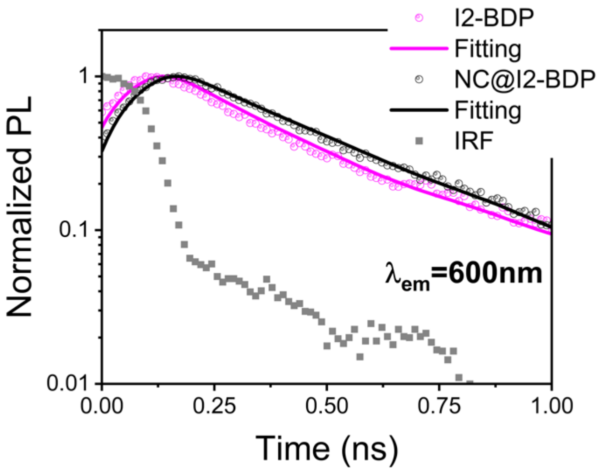

Figure S7. PL decay traces of I2-BDP (pink curve) and NC@I2-BDP (black curve) in toluene monitored at 600 nm where only I2-BDP is emissive under 450 nm pulsed laser excitation (9.8 MHz). The instrument response function (IRF) was ca. 150 ps calculated at FWHM.

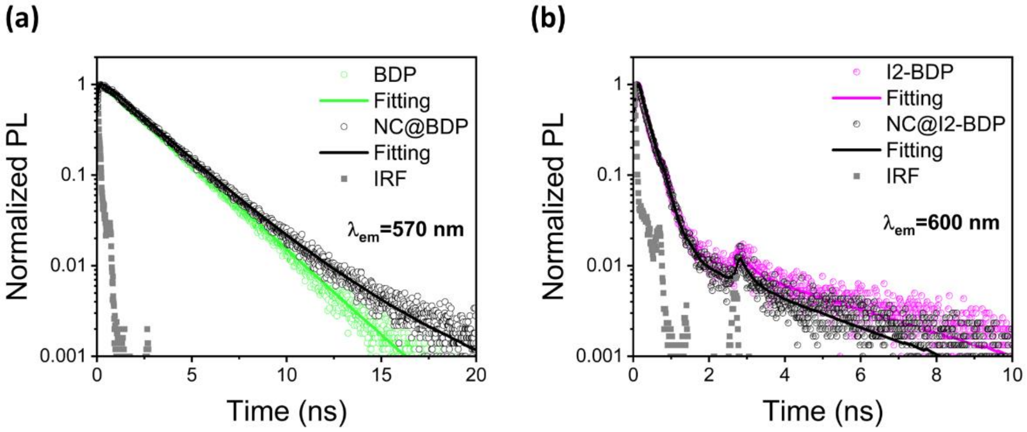

Figure S8. PL decay traces of (a) BDP and (b) I2-BDP in toluene along with those in their corresponding nanohybrids monitored at 570 nm and 600 nm, respectively. Excitation at 515 nm and 550 nm with a pulsed laser source (9.8 MHz) was used for (a) and (b), respectively. The instrument response function (IRF) was ca. 150 ps calculated at FWHM.

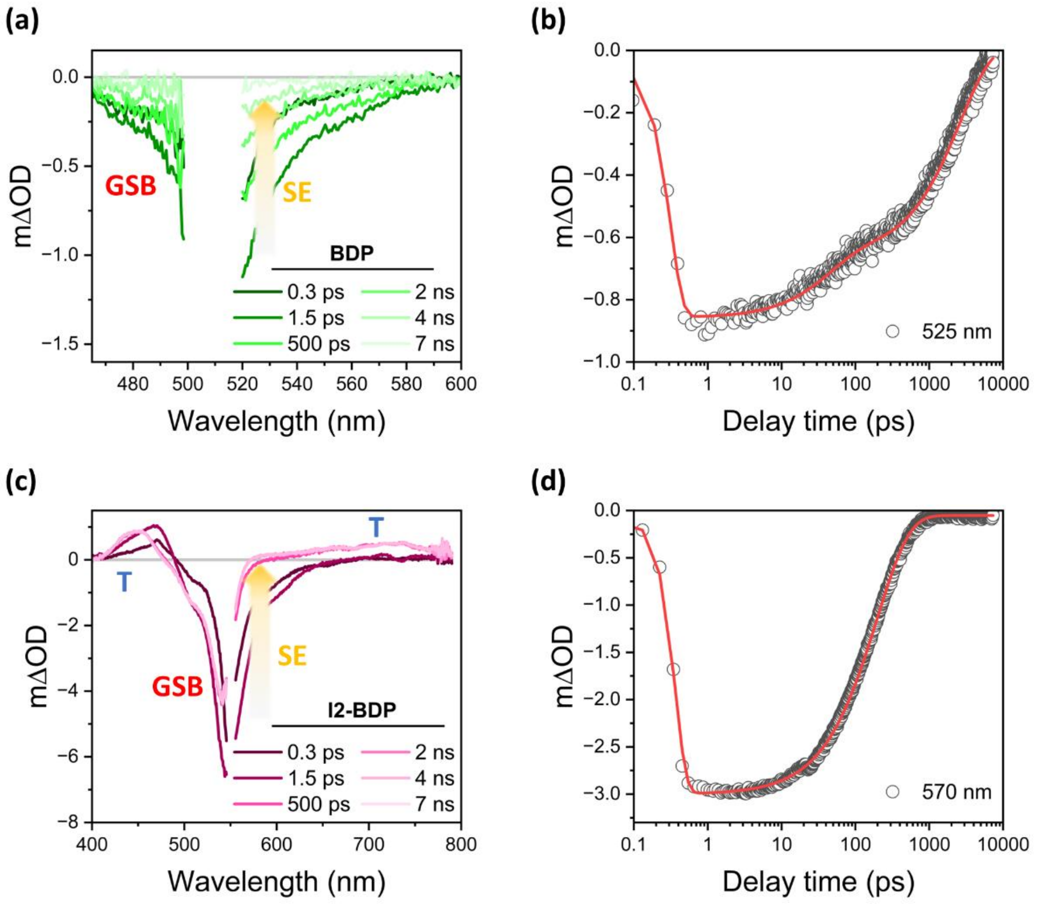

Figure S9. fs-TA properties of BODIPY dyes in toluene. TA spectra at indicated delay times for BDP (a) and I2-BDP (c) along with their corresponding fluorescence kinetics for BDP (b) and I2-BDP (d), respectively. Solid lines indicate fitting curve. The removed region in spectra is due to some noise caused by pump excitation.

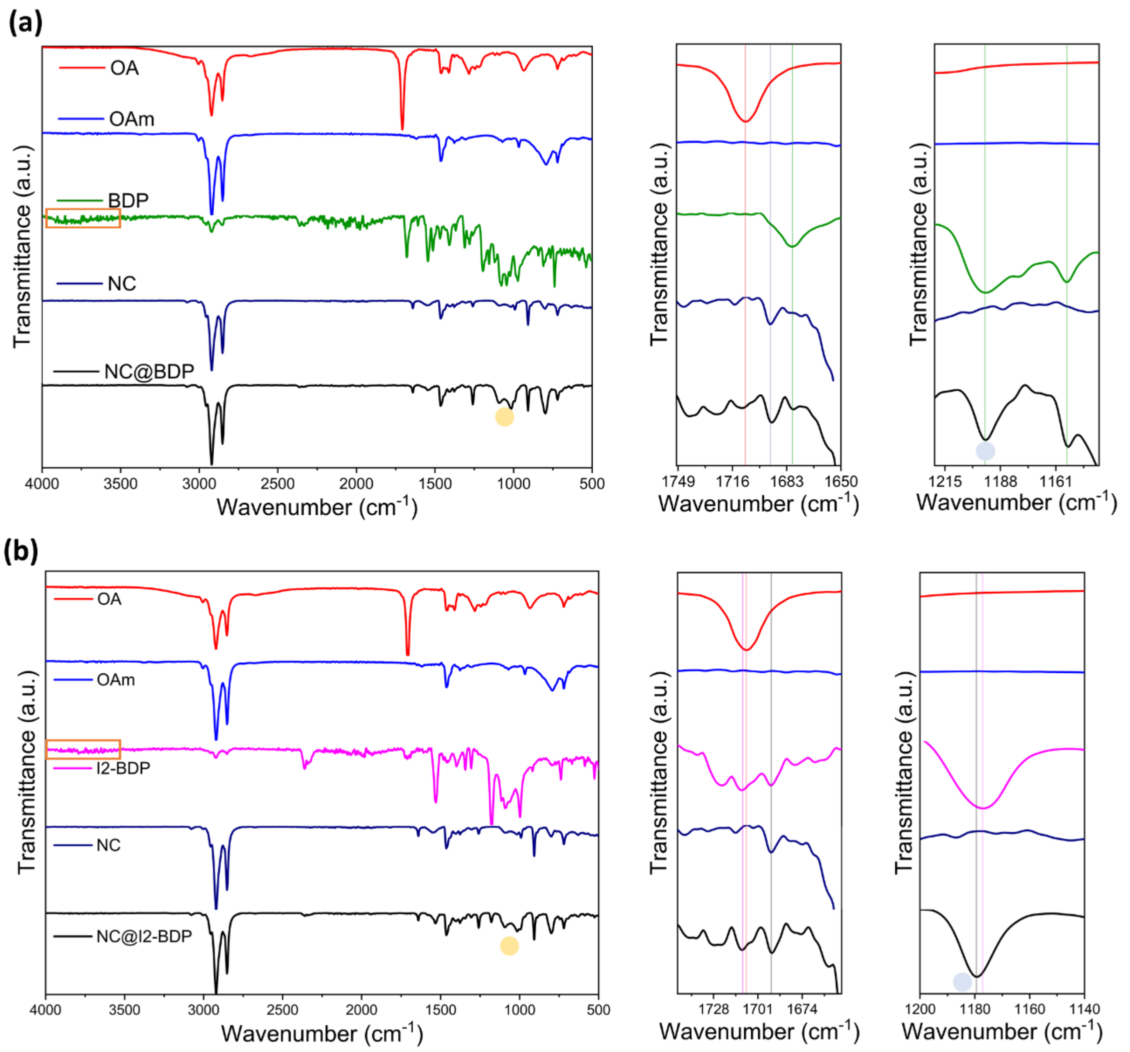

Figure S10. Fourier-transform infrared (FTIR) attenuated total reflection (ATR) spectra of (a) NC@BDP and (b) NC@I2-BDP along with their corresponding controls (NC and dye), OA and OAm. Enlarged view in the region of interest for (a) 1749-1650 cm^{-1} and 1218-1140 cm^{-1} and (b) 1749-1650 cm^{-1} and 1200-1140 cm^{-1}.

Observations on FTIR

The attachment of both BODIPY dyes to NC surface by (i) the disappearance of the O-H stretching vibration of both dyes in NCs@dye nanohybrids (orange box) which is a clear indication of the anchoring to lead cations on the NC surface. (ii) The widening of the bands of NC@dye nanohybrids (yellow circle) when compared to pristine NC clearly means these bands are powered by the BODIPY surrounding. (iii) The sharpness in the band marked with a blue circle.

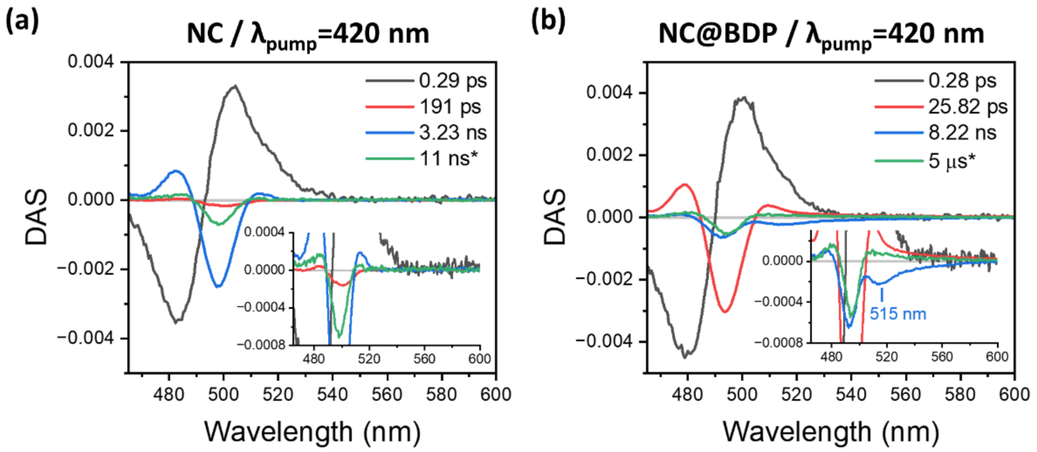

Figure S11. Decay associated spectra (DAS) obtained by GA for (a) pristine NC and (b) NC@BDP nanohybrids in toluene under 420 nm pump excitation.

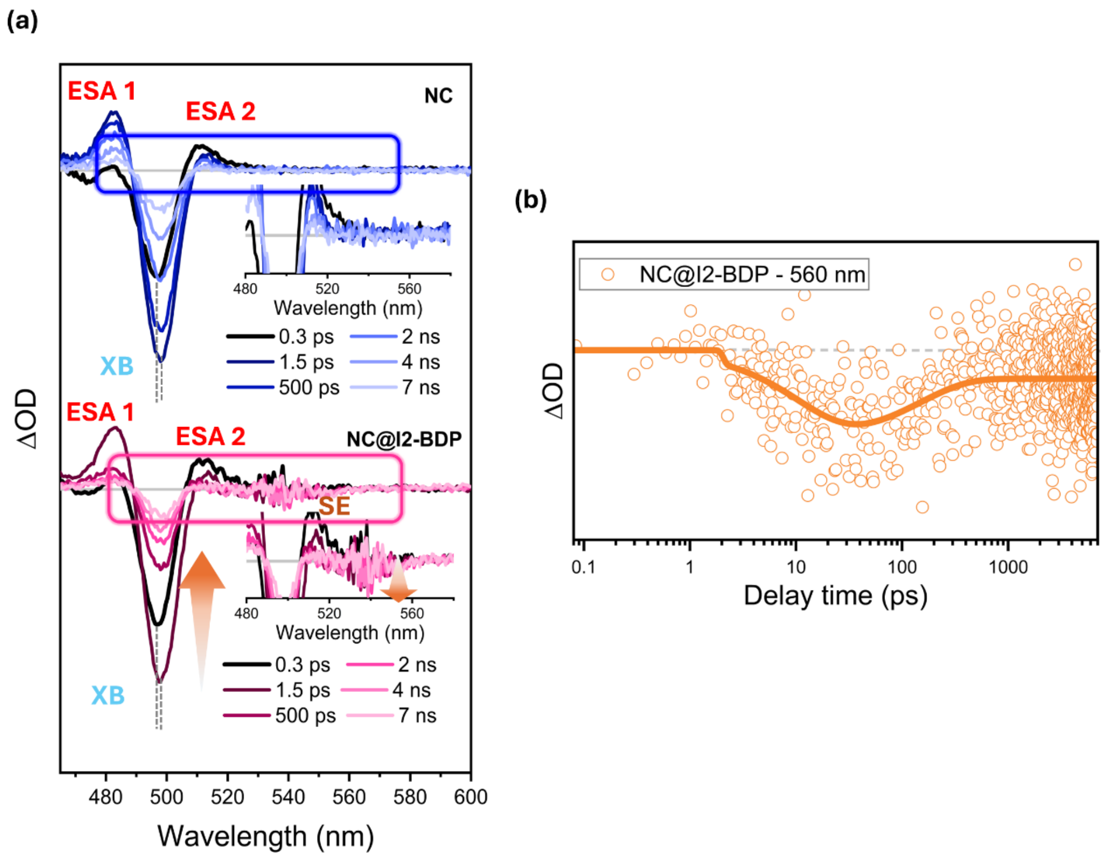

Figure S12. (a) TA spectra for NC (top) and NC@I2-BDP (bottom) at indicated delay times. Inset: enlarged TA region. (b) Kinetic trace of NC@I2-BDP nanohybrid probed at 560 nm (orange hollow circle) and best multiexponential fitting (orange solid line).

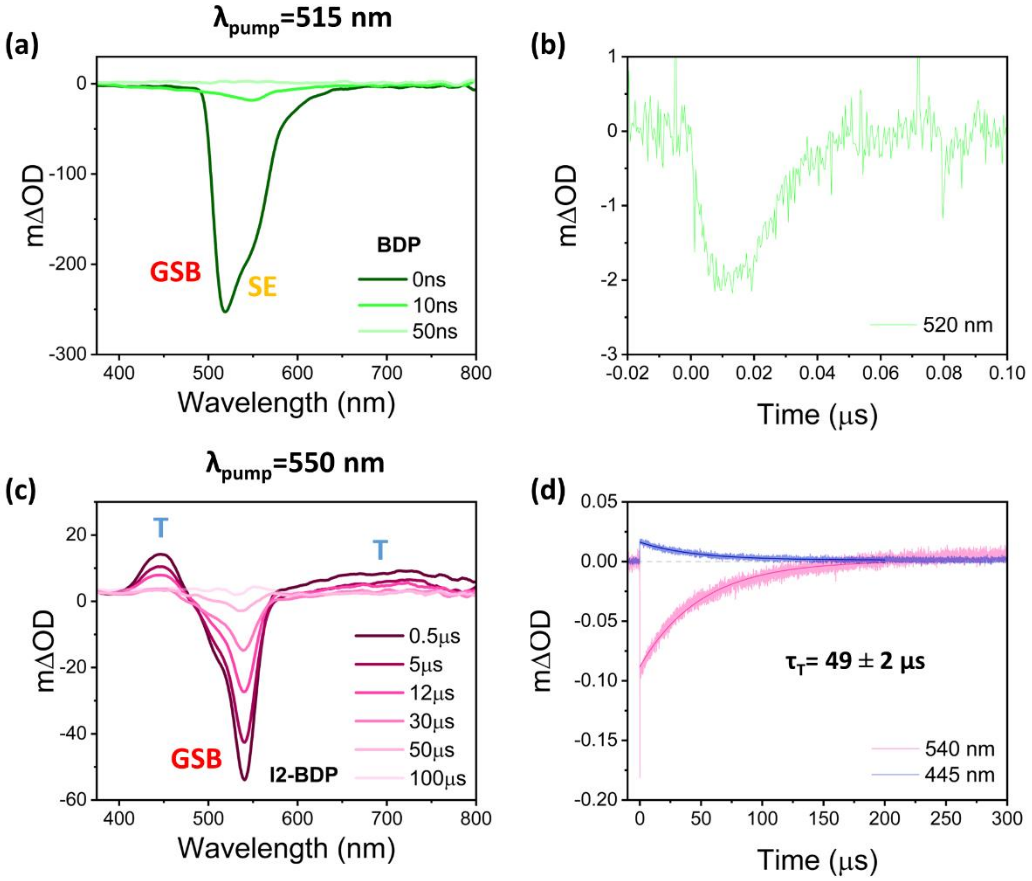

Figure 13. ns-TA properties of BODIPY dyes in toluene. TA spectra at indicated delay times for BDP (a) and I2-BDP (c) along with their corresponding kinetics at indicated probe wavelength for BDP (b) and I2-BDP (d), respectively. Solid lines in panel (d) describe the fitting curve.

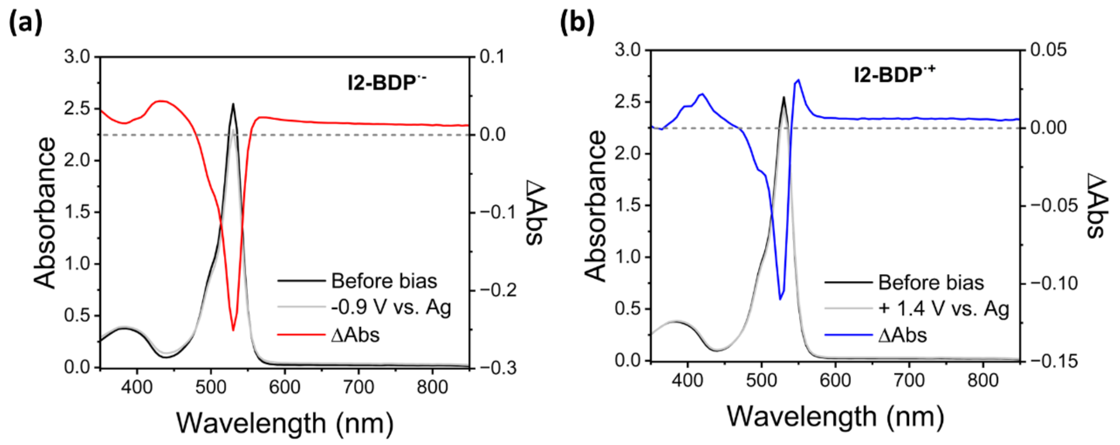

Figure S14. UV-vis absorption spectra (before and after applying a bias) and the difference spectra of I2-BDP dye for cathodic potentials (a) and anodic potentials (b).

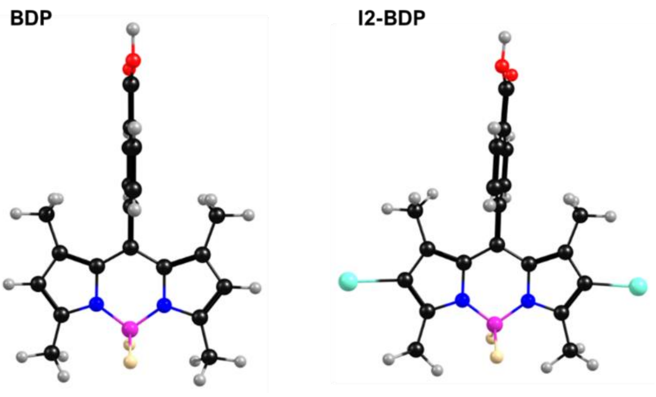

Figure S15. Optimized structure of the BDP (left) and I2-BDP (right) in toluene.

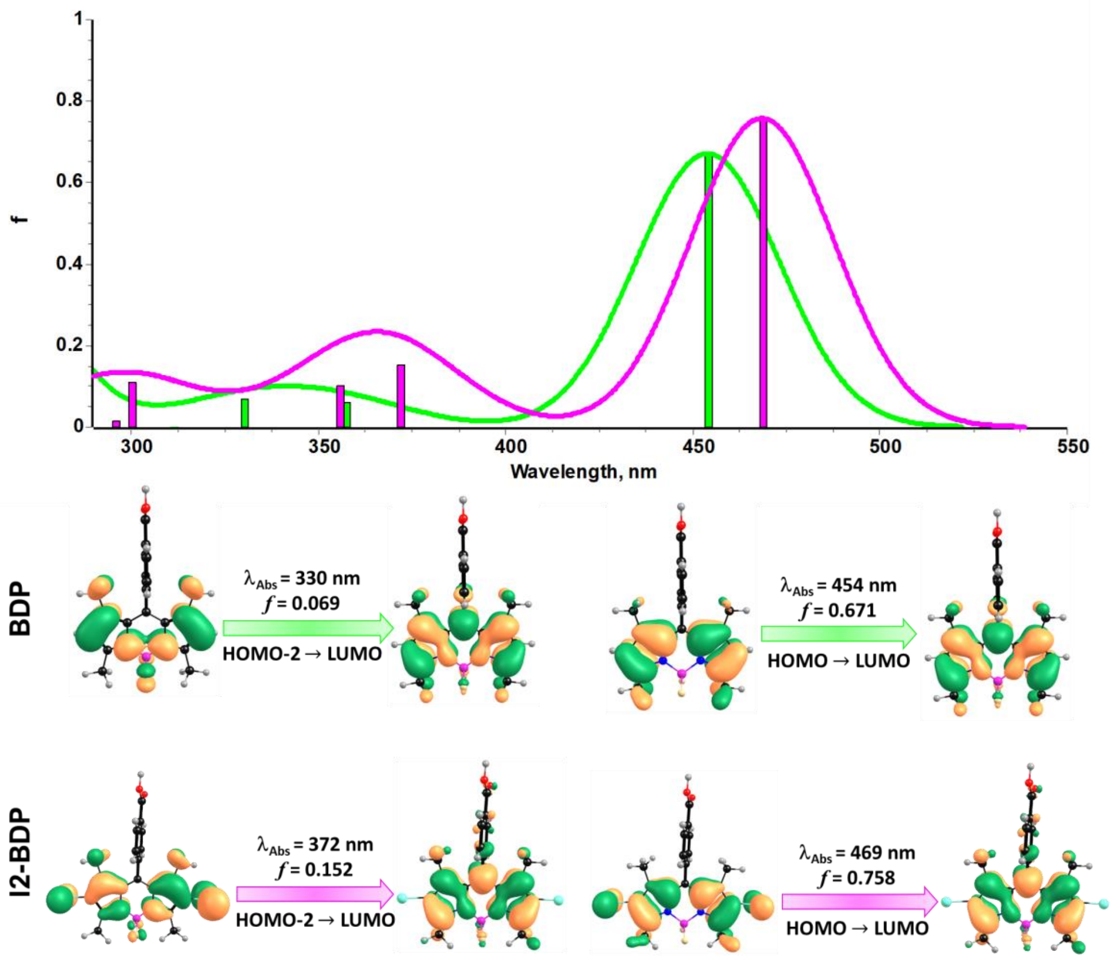

Figure S16. TDDFT electronic absorption spectra and natural transition orbitals (NTOs) of the BDP (green curve) and I2-BDP (pink curve) for absorption in toluene.

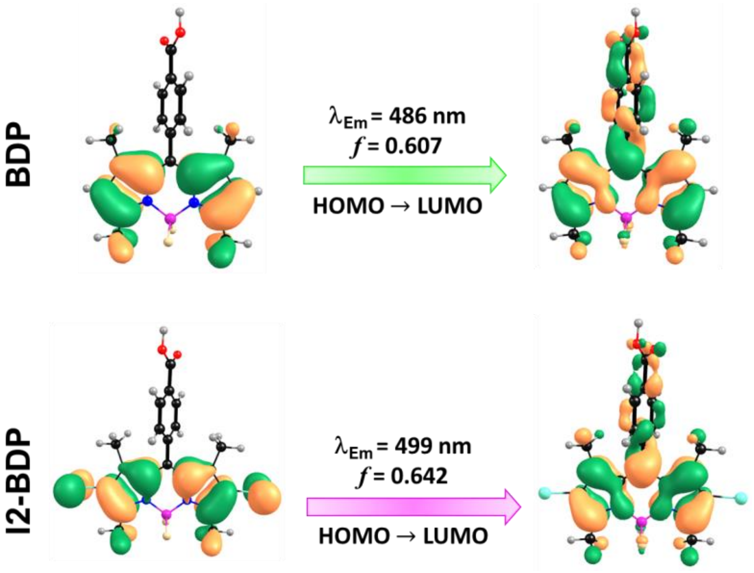

Figure S17. Natural transition orbitals (NTOs) of the BDP (up) and I2-BDP (bottom) for emission in toluene.

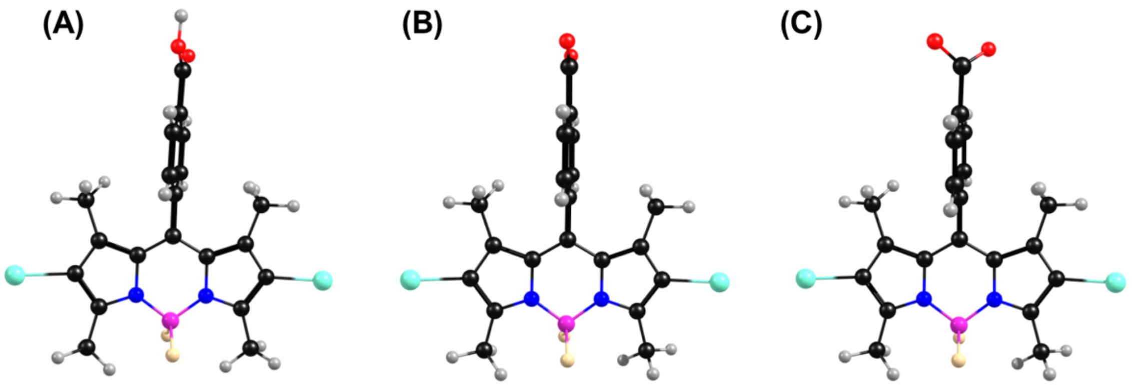

Figure S18. Optimized structure of the I2-BDP in (a) neutral, (b) radical anion and (c) radical cation forms in toluene.

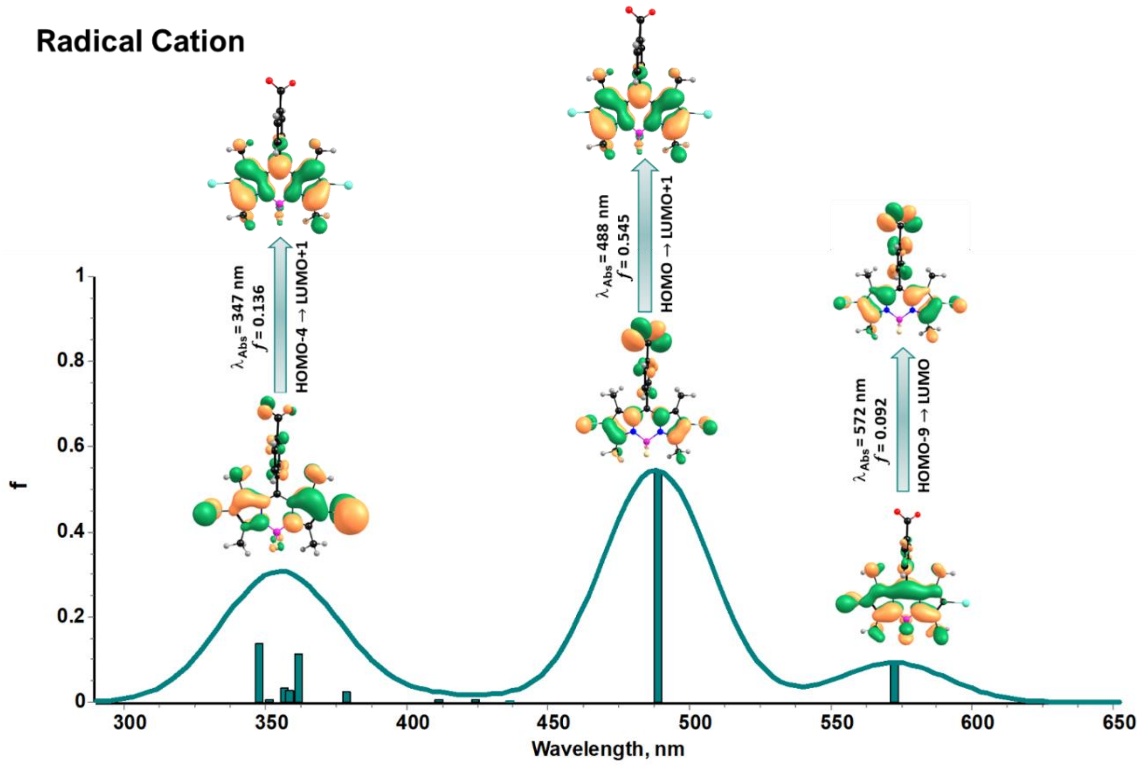

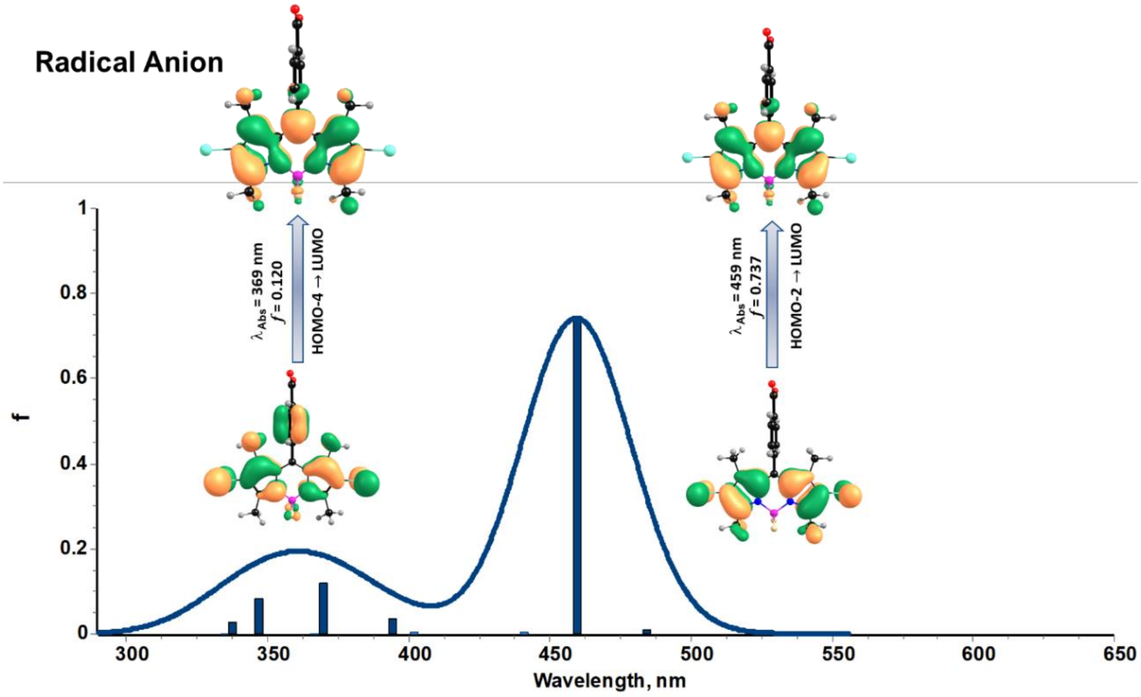

Figure S19. TDDFT electronic absorption spectra and surfaces of NTOs of probe I2-BDP in radical cation and radical anion forms in toluene.

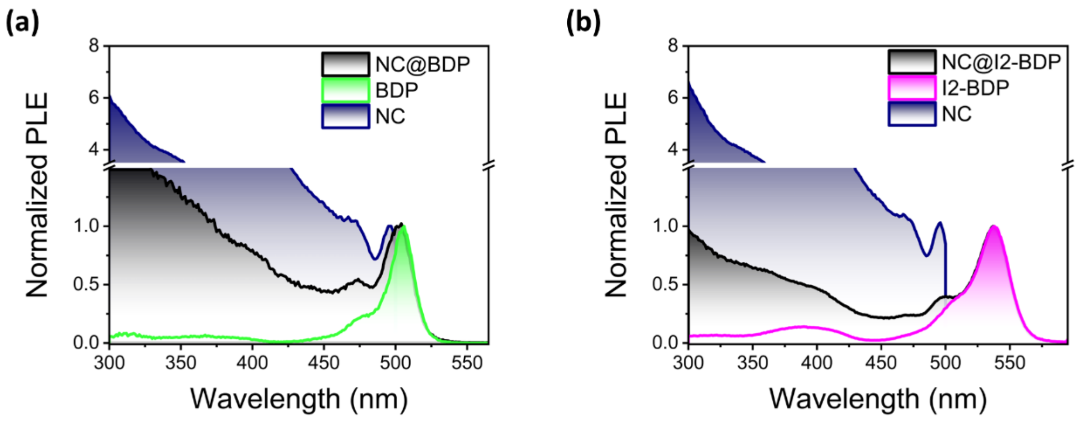

Figure S20. Steady-state normalized photoluminescence excitation (PLE) properties of (a) NC@BDP and (b) NC@I2-BDP, respectively along with their corresponding controls in toluene. Emission wavelength is 570 nm (BDP and NC@BDP) and 502 nm (NC) for (a), and 600 nm (I2-BDP and NC@I2-BDP) and 502 nm (NC) for (b).

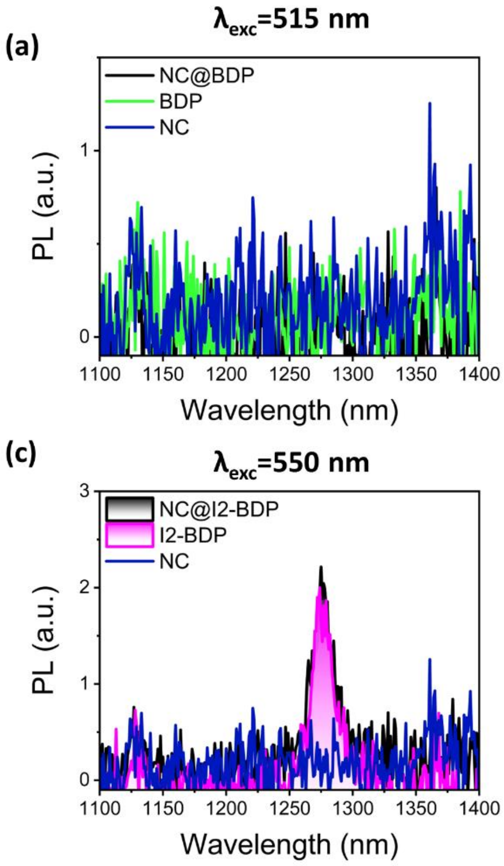

Figure S21. Singlet oxygen phosphorescence ($^1O_2$) measurements of NC@BDP nanohybrids in toluene along with their controls recorded upon excitation at (a) 515 nm and (b) 420 nm. The same for NC@I2-BDP nanohybrids at (c) 550 nm and (d) 450 nm. All cuvettes were purged with O_2 for 5 min.

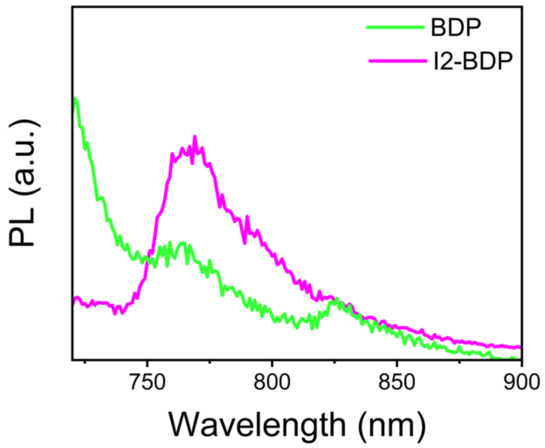

Figure S22. Phosphorescence spectra of BDP (green curve) and I2-BDP (pink curve) dyes in toluene under 515 nm and 550 nm excitation, respectively, with a pulsed xenon lamp with 100 kHz repetition rate. The spectra were registered at 77 K. The absorbance at excitation wavelengths was 0.3.

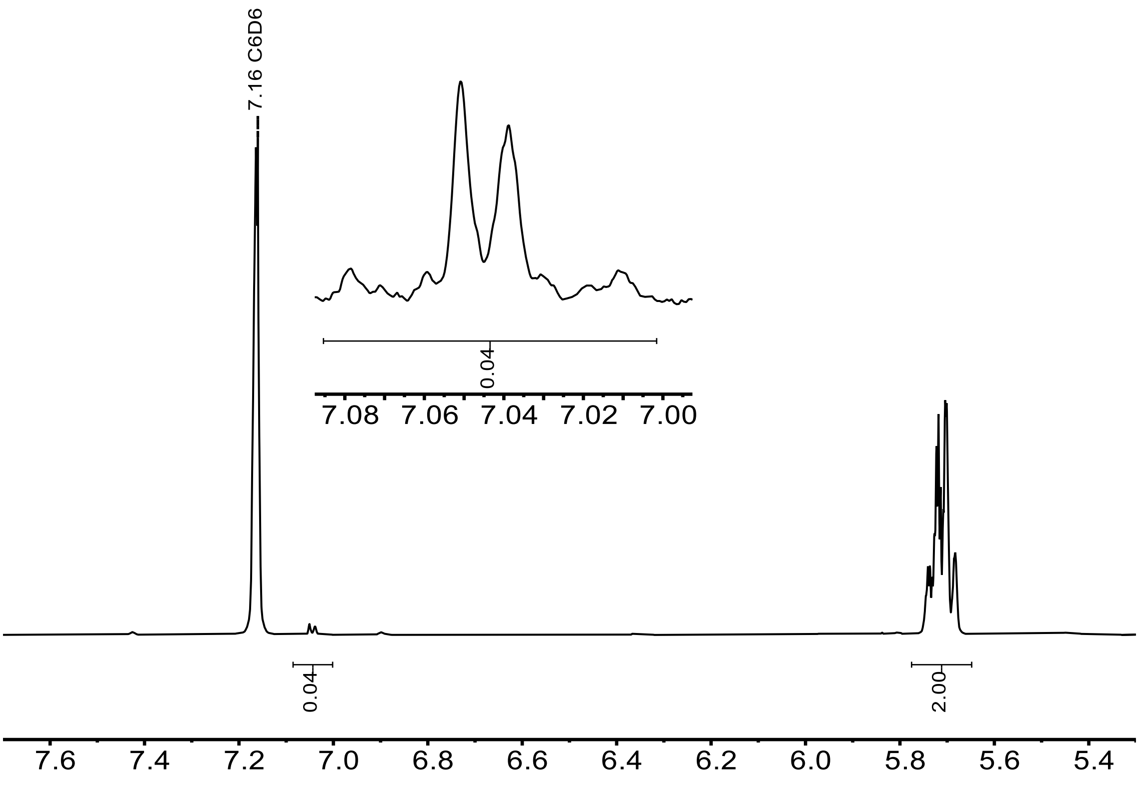

Figure S23. ^{1}H-NMR spectrum of purified α-terpinene in C_6D_6. It has been purified through silica column chromatography (hexane) giving rise to a 99% and 1% purity of α-terpinene and p-cymene, respectively.

References

(1) Protesescu, L.; Yakunin, S.; Bodnarchuk, M. I.; Krieg, F.; Caputo, R.; Hendon, C. H.; Yang, R. X.; Walsh, A.; Kovalenko, M. V. Nanocrystals of Cesium Lead Halide Perovskites ($CsPbX_3$, X = Cl, Br, and I): Novel Optoelectronic Materials Showing Bright Emission with Wide Color Gamut. *Nano Letters* **2015,** *15* (6), 3692.

(2) Li, W.; Si, L.; Liu, Z.; Zhao, Z.; He, H.; Zhu, K.; Moore, B.; Cheng, Y.-B. Fluorene functionalized porphyrins as broadband absorbers for TiO_2 nanocrystalline solar cells. *Journal of Materials Chemistry A* **2014,** *2* (33), 13667.

(3) Kolemen, S.; Bozdemir, O. A.; Cakmak, Y.; Barin, G.; Erten-Ela, S.; Marszalek, M.; Yum, J.-H.; Zakeeruddin, S. M.; Nazeeruddin, M. K.; Grätzel, M.et al. Optimization of distyryl-Bodipy chromophores for efficient panchromatic sensitization in dye sensitized solar cells. *Chemical Science* **2011,** *2* (5), 949.

(4) Snellenburg, J. J.; Laptenok, S.; Seger, R.; Mullen, K. M.; van Stokkum, I. H. M. Glotaran: A Java-Based Graphical User Interface for the R Package TIMP. *Journal of Statistical Software* **2012,** *49* (3), 1

(5) Lakowicz, J. R. *Principles of Fluorescence Spectroscopy*; Springer US, 2007.

(6) Schmidt, M. W.; Baldridge, K. K.; Boatz, J. A.; Elbert, S. T.; Gordon, M. S.; Jensen, J. H.; Koseki, S.; Matsunaga, N.; Nguyen, K. A.; Su, S.et al. General atomic and molecular electronic structure system. *Journal of Computational Chemistry* **1993,** *14* (11), 1347.
